# Targeting Tropomyosin Receptor Kinase in Cutaneous CYLD Defective Tumors With Pegcantratinib: The TRAC Randomized Clinical Trial

**DOI:** 10.1001/jamadermatol.2018.1610

**Published:** 2018-06-27

**Authors:** Marina Danilenko, Elaine Stamp, Deborah D. Stocken, Akhtar Husain, Monique Zangarini, Amy Cranston, Robert Stones, Naomi Sinclair, Kirsty Hodgson, Susan A. Bowett, David Roblin, Silvio Traversa, Ruth Plummer, Gareth Veal, James A. A. Langtry, Alan Ashworth, John Burn, Neil Rajan

**Affiliations:** 1 Institute of Genetic Medicine, Newcastle University, Newcastle upon Tyne, United Kingdom; 2 Biostatistics Research Group, Institute of Health and Society, Newcastle University, Newcastle upon Tyne, United Kingdom; 3 Department of Dermatology, Royal Victoria Infirmary, Newcastle, United Kingdom; 4 Northern Institute for Cancer Research, Newcastle University, Newcastle upon Tyne, United Kingdom; 5 Newcastle Clinical Trials Unit, Newcastle University, Newcastle upon Tyne, United Kingdom; 6 The Francis Crick Institute, London, United Kingdom; 7 Sienna Biopharmaceuticals, Inc, Colleretto Giacosa, Italy; 8 University of California San Francisco Helen Diller Family Comprehensive Cancer Center, San Francisco

## Abstract

**Importance:**

There are no medical interventions for the orphan disease CYLD cutaneous syndrome (CCS). Transcriptomic profiling of CCS skin tumors previously highlighted tropomyosin receptor kinases (TRKs) as candidate therapeutic targets.

**Objective:**

To investigate if topical targeting of TRK with an existing topical TRK inhibitor, pegcantratinib, 0.5% (wt/wt), is safe and efficacious in CCS.

**Design, Setting, and Participants:**

A phase 1b open-label safety study, followed by a phase 2a within-patient randomized (by tumor), double-blind, placebo-controlled trial (the Tropomyosin Receptor Antagonism in Cylindromatosis [TRAC] trial). The setting was a single-center trial based at a tertiary dermatogenetics referral center for CCS (Royal Victoria Infirmary, Newcastle, United Kingdom). Patients who had germline mutations in *CYLD* or who satisfied clinical diagnostic criteria for CCS were recruited between March 1, 2015, and July 1, 2016.

**Interventions:**

In phase 1b, patients with CCS applied pegcantratinib for 4 weeks to a single skin tumor. In phase 2a, allocation of tumors was to either receive active treatment on the right side and placebo on the left side (arm A) or active treatment on the left side and placebo on the right side (arm B). Patients were eligible if they had 10 small skin tumors, with 5 matched lesions on each body side; patients were randomized to receive active treatment (pegcantratinib) to one body side and placebo to the other side once daily for 12 weeks.

**Main Outcomes and Measures:**

The primary outcome measure was the number of tumors meeting the criteria for response in a prespecified critical number of pegcantratinib-treated tumors. Secondary clinical outcome measures included an assessment for safety of application, pain in early tumors, and compliance with the trial protocol.

**Results:**

In phase 1b, 8 female patients with a median age of 60 years (age range, 41-80 years) were recruited and completed the study. None of the participants experienced any adverse treatment site reactions. Three patients reported reduced pain in treated tumors. In phase 2a (15 patients [13 female; median age, 51 years], with 150 tumors), 2 tumors treated with pegcantratinib achieved the primary outcome measure of response compared with 6 tumors treated with placebo. The primary prespecified number of responses was not met. The incidence of adverse events was low.

**Conclusions and Relevance:**

In this study, pegcantratinib, 0.5% (wt/wt), applied once daily appeared to be well tolerated and to penetrate the tumor tissue; however, the low tumor drug concentrations demonstrated are likely to account for the lack of response. Dose-escalation studies to assess the maximal tolerated dose may be beneficial in future studies of CCS.

**Trial Registration:**

isrctn.org Identifier: ISRCTN75715723

## Key Points

**Question:**

Can targeting tropomyosin receptor kinase with an existing topical kinase inhibitor, pegcantratinib, 0.5% (wt/wt), reduce cutaneous cylindroma tumor volume more than placebo?

**Findings:**

In this phase 2 clinical trial that included 150 tumors from 15 patients with CYLD cutaneous syndrome, pegcantratinib-treated tumors did not achieve the primary outcome of response. Molecular analyses of biopsy material demonstrated drug penetration; however, drug concentrations achieved were inadequate to abrogate tropomyosin receptor kinase signaling in CYLD cutaneous syndrome tumors.

**Meaning:**

These findings indicate that further studies should examine dose-escalation of pegcantratinib in these patients.

## Teaser

This phase 2 randomized clinical trial investigates if topical targeting of topical tropomyosin receptor kinase (TRK) with an inhibitor, pegcantratinib, is safe and efficacious in treating tumors in patients with CYLD cutaneous syndrome.

## Introduction

Patients with germline mutations in the tumor suppressor gene *CYLD* (OMIM 605018) develop multiple, disfiguring, hair follicle tumors on the head and neck. This condition, named CYLD cutaneous syndrome (CCS) (also known as Brooke-Spiegler syndrome [605041]), is rare; however, the effect on individual patients can be devastating, with up to 1 in 4 mutation carriers requiring complete surgical removal of the scalp.^1^ These patients also have numerous tumors on the trunk that are symptomatic with pain and prone to ulceration and bleeding. Tumors have a predilection to develop on the external ear and in the ear canal, resulting in conductive deafness. An additional predisposed site is pubic skin, associated with sexual dysfunction. These patients require repeated surgery to control tumor burden and lifelong monitoring of tumors, for which malignant transformation is infrequently reported. Radiotherapy is of limited benefit and carries the attendant risk of further tumor induction within the treatment field, as well as malignant transformation of treated tumors.^2^ To date, there are no effective medical alternatives to treat this rare orphan disease.^1^

Tropomyosin receptor kinase (TRK) was discovered as a candidate drug target after a search for targetable kinases in inherited CCS tumors using an unbiased genetic approach.^3^ In the absence of any personalized, randomized, placebo-controlled trials for CCS, transcriptomics of human CCS tumors was used to aid the discovery of oncogenic kinases that were overexpressed and targetable.^3^ Alterations in DNA and differences in RNA expression of fresh, snap-frozen tumors compared with adjacent, unaffected skin were characterized and led to 2 key findings.^3^ First, the genetic changes in these tumors were limited, with loss of heterozygosity at the *CYLD* locus being the only recurrent genetic change seen. This homogeneity implied that a targetable kinase discovered on this genetic background might have an effect on most tumors. Second, overexpression of TRKs selectively in tumor cells was identified, with overexpression of TRKB and TRKC in almost all tumors examined. Although the mechanism by which loss of CYLD function results in perturbation of TRK homeostasis is not fully understood, TRK signaling has been shown to confer a survival advantage to tumor cells by increasing resistance to apoptosis and cell proliferation.^4,5,6^ This is pertinent because new-generation oral TRK inhibitors are now available, targeting cancers that overexpress TRK after gene rearrangement.^7^

Pegcantratinib (previously CT327) is a potent TRKA inhibitor with activity against TRKB and TRKC. It is a topical investigational medicinal product developed by Creabilis SA (now Sienna Biopharmaceuticals, Inc) for the treatment of inflammatory dermatoses, such as psoriasis and concomitant pruritus. Significant and clinically meaningful reduction in psoriatic pruritus was reported previously, which occurs via a TRKA-dependent mechanism, in patients treated with topical pegcantratinib, 0.5% (wt/wt).^8^ To our knowledge, the present trial is the first to investigate pegcantratinib use in patients with CCS; however, the drug has been given to 36 healthy volunteers and 336 patients in the clinical trials to date^8,9^ and was well tolerated at concentrations up to 0.5% wt/wt. These safety data supported the application of 0.5% wt/wt concentration in our patients.

We designed an early-phase exploratory trial to investigate if delivering pegcantratinib to CCS tumors might represent a safe and feasible noninvasive treatment. This study is of translational relevance because *Cyld* transgenic mice fail to recapitulate the human CCS tumor phenotype,^1^ making the study of in vivo effects of TRK inhibition in humans with a topical intervention a necessary approach that overcomes this limitation. Furthermore, we leveraged the multiplicity of skin tumors in these patients to develop a statistically powered study, which is challenging in rare disease. The trial also offered the opportunity to gain novel insights into CCS, including its natural history, the rate of growth of early tumors, the frequency of pain in small tumors, and the effect of disease on patients’ quality of life.

## Methods

### Study Design

This was an investigator-initiated 2-part single-center phase 1b/2a exploratory trial (Tropomyosin Receptor Antagonism in Cylindromatosis [TRAC])^10^ to investigate the safety and preliminary efficacy of topical pegcantratinib in patients with inherited CYLD defective skin tumors. Regulatory approvals were sought and obtained from a human participants ethics review board (National Research Ethics Service Committee North East-Tyne and Wear [14/NE/1080;06/1059]) and the Medicines Health Regulatory Authority (EudraCT:2014-001342-21), and the trial was registered (ISRCTN75715723) and the trial protocol can be found in Supplement 1. All recruited patients provided written informed consent.

Phase 1b was an open-label study to determine the short-term safety and tolerability of applying pegcantratinib, 0.5% (wt/wt) to a preselected single skin tumor (<30 mm in diameter) in a *CYLD* mutation carrier that was scheduled for routine excision. The treated site was clinically assessed using a modified version of the Draize score test for signs of local site reaction,^11^ a measure of skin inflammation, after 4 weeks. The lack of reactions (modified Draize score of ≤3) in at least 5 of 8 treated tumors allowed the trial to progress to phase 2a.

**Figure 1.**
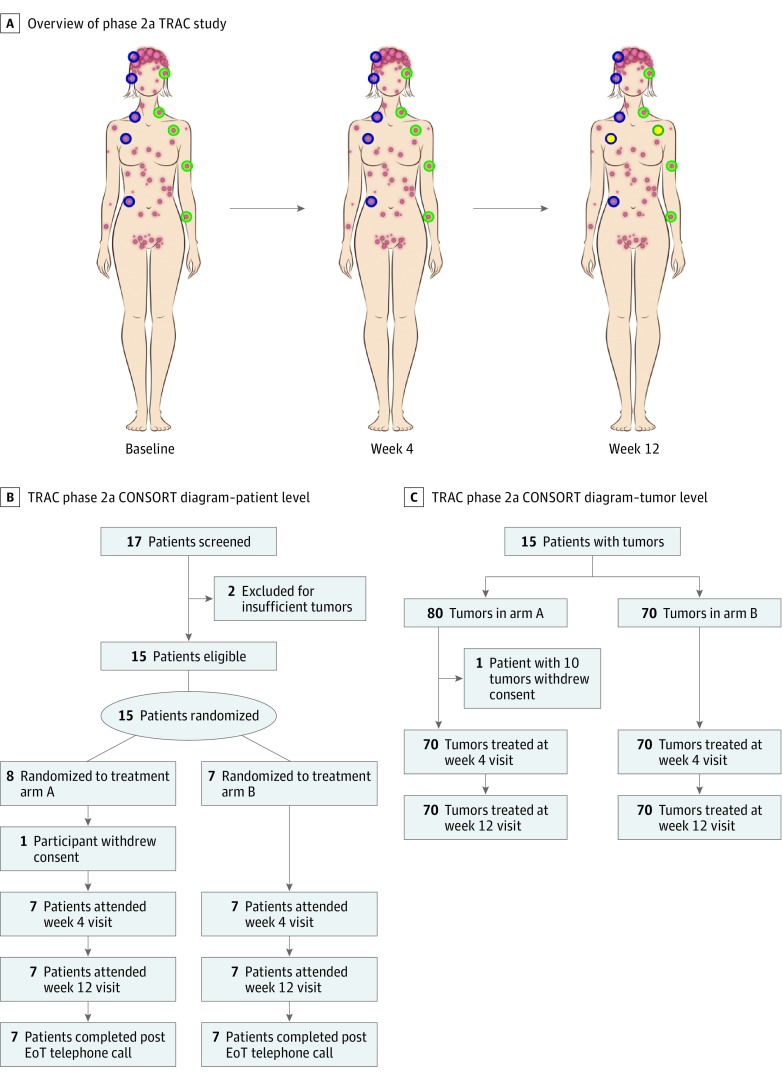
An Overview of TRAC Phase 2a and the CONSORT Diagram Indicating Patient Recruitment and Tumor Assignment to Active and Placebo Treatments A, Baseline volume measurements were made of 10 tumors, 5 on the left matched with 5 on the right. In the example shown, the masked patient then applied active treatment (blue circles) to the patient’s right side and placebo to the patient’s left side (green circles). Four weeks later, volume measurements were taken of all 10 tumors. At the final visit at 12 weeks, volume measurements were taken of all 10 tumors, and a lesion on each side was biopsied for molecular analyses (yellow filled circles). B, CONSORT diagram indicating patient screening, recruitment, and dropout, with 14 patients completing phase 2a. C, Tumor-level CONSORT data on allocation of tumors to either receive active treatment on the right side and placebo on the left side (arm A) or active treatment on the left side and placebo on the right side (arm B). CONSORT indicates Consolidated Standards of Reporting Trials; EoT, end of trial; TRAC, Tropomyosin Receptor Antagonism in Cylindromatosis (TRAC).

Phase 2a was a within-patient (and by tumor) randomized, double-blind, placebo-controlled trial (Figure 1A). The setting was a single-center trial based at a tertiary dermatogenetics referral center for CCS (Royal Victoria Infirmary, Newcastle, United Kingdom). We aimed to recruit 15 to 20 patients, each with 10 eligible small tumors (<10 mm in diameter, with 5 matched lesions on each body side), with a target of 150 tumors treated and assessed for 12 weeks. Volume measurements and tumor pain assessments were taken at baseline, week 4, and week 12 by a masked assessor (M.D. and N.R.). At the 12-week (final) visit, one randomly selected recruited tumor was biopsied (punch biopsy specimen 4-6 mm in diameter) from each body side and snap frozen for molecular analyses.

Phase 2a was designed using single-arm Fleming-A’Hern early-phase methods^12^ to investigate whether pegcantratinib could be a treatment suitable for further investigation in this patient group. Seventy-five tumors recruited in the experimental arm provide 3.4% type I errors and 10.8% type II errors when decision making, based on observing a minimum number of responses. The trial recruited an equal number of placebo-treated tumors to provide an unbiased benchmark.

### Randomization and Masking

In phase 2a, randomization was at the individual patient level, with active treatment randomized to tumors on one side of the body and placebo on the opposite side. Application was performed once daily for 12 weeks. Randomization was performed centrally by the Newcastle Clinical Trials Unit (Newcastle University, Newcastle upon Tyne, United Kingdom) internet-accessed secure web-based system. Five small tumors matched for size were selected on each side of the patient and marked with an ink dot and numbered.

Phase 2a patients and investigators (M.D. and N.R.) were masked to the treatment allocation. Those responsible for tumor volume measurements, histology assessments, and molecular analyses (M.D., A.H., M.Z., R.S., G.V., and N.R.) were also masked to the treatment allocation.

### Procedures

In phase 1b, open-label, active trial medication containing pegcantratinib, 0.5% (wt/wt) was provided as ointment in glass vials. Treatment was applied once daily for 4 weeks.

In phase 2a, participants treated tumors only on half of the body with pegcantratinib, 0.5% (wt/wt) and tumors only on the other half of the body with placebo (ie, sham ointment with emollient properties similar to those of pegcantratinib) once daily according to the randomization allocation for a 12-week period. A once-daily application was chosen as feasible for this period after consultation with a group of patients with CCS. Patients were provided with a spatula to standardize dosage. During clinician-supervised instruction (N.R.) of the application of the first dose, generous coverage of each tumor was confirmed.

### Outcome Measures

#### Primary

In phase 1b, the primary outcome measure was the number of severe treated skin site reactions. These were defined as a modified Draize score of 4 or higher after 4 weeks of treatment.

In phase 2a, the primary outcome was the number of tumors meeting the criteria for response, namely, a reduction in volume by 30% from baseline seen in a critical number (n = 12) of pegcantratinib-treated tumors. Tumor volume measurements were made using a validated stereoscopic skin tumor imaging platform (LifeViz Micro; QuantifiCare SA).^13^

#### Secondary

Both phases opportunistically assessed the effect of CCS disease using patient-reported quality-of-life tools (the Dermatology Life Quality Index [DLQI]^14^ and the EuroQol–5 Dimension [EQ-5D]^15^). Adverse events were graded as mild, moderate, or severe within the treatment period; compliance was as reported in a patient diary recorded throughout the treatment period; and patient acceptability to trial treatment was assessed by a questionnaire at the end of treatment.

Additional secondary measures for phase 2a were change in tumor volume from baseline (prerandomization) to 12 weeks assessed by a tumor volume measuring device; patient-reported pain using a trial-specific questionnaire at 0, 4, and 12 weeks; and expression of targets of TRK signaling in tumor biopsy specimens as determined by quantitative polymerase chain reaction and immunohistochemistry. Additional methods are available in eMethods in Supplement 2.

## Results

**Figure 2.**
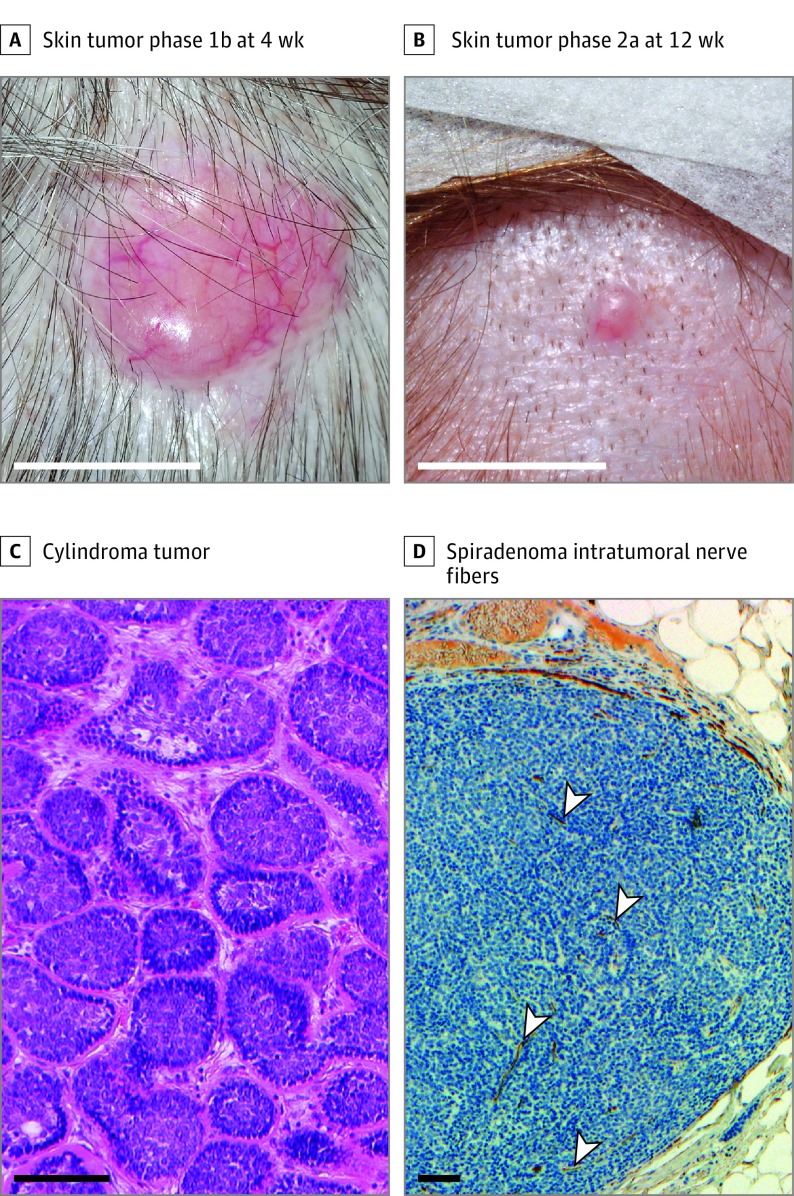
Skin Tumors in the Tropomyosin Receptor Antagonism in Cylindromatosis (TRAC) Study A, A large skin tumor that was recruited to phase 1b is shown after 4 weeks of treatment with pegcantratinib, 0.5% (wt/wt), with no cutaneous inflammation noted. B, A small skin tumor that was recruited to phase 2a is shown after 12 weeks of treatment with pegcantratinib, with no cutaneous inflammation noted. C, Shown is histology of a cylindroma, demonstrating tumor cells arranged in cylinders (hematoxylin-eosin, original magnification ×20). D, Shown is immunohistochemical staining of spiradenoma with neurofilament, a marker of nerve fibers, indicating frequent innervation of tumors, both at the edge and within (arrowheads) the tumor mass of cells (neurofilament stain, original magnification ×10). White scale bars indicate 10 mm; black scale bars indicate 100 μm.

Between March 1, 2015, and July 1, 2016, a total of 23 patients who had germline mutations in *CYLD* or who satisfied clinical diagnostic criteria for CCS were recruited. In phase 1b, 8 female patients with a median age of 60 years (age range, 41-80 years) were recruited and completed the study. Each patient had a single large tumor selected for inclusion, and these tumors had a mean base diameter of 10.6 mm (range, 5-18 mm). None of the 8 patients developed any treatment site reactions, and all had a modified Draize score of 0 (Figure 2A). Compliance with the treatment application was excellent, with 98.7% of intended applications administered. All patients reported that they would use the ointment if it was available as a treatment. Notably, 3 patients reported that their tumors had become less painful. Adverse event reporting identified 1 patient who developed shingles away from the treated site, and this was reported as mild and deemed unrelated to treatment. These safety and acceptability data allowed for progression to phase 2a.

In phase 2a, 15 patients with CCS were recruited, of whom 7 had participated in phase 1b. In phase 2a, allocation of tumors was to either receive active treatment on the right side and placebo on the left side (arm A) or active treatment on the left side and placebo on the right side (arm B). Thirteen patients were female, and the median age of participants was 51 years (age range, 37-74 years). Patients had a range of comorbidities and medications (eTable 1 in Supplement 2). Each patient had at least 10 eligible tumors that allowed for matching for size, with 5 selected on the left side of the patient and 5 selected on the right side (eTable 2 in Supplement 2). These small tumors (mean, 4.23 mm in diameter) selected against predefined criteria that were distinct from phase 1b (Figure 2B) because we wanted to study tumors before they reached a size when they were typically excised. Tumor characteristics indicated adequate matching in both arms of the trial (eTable 3 in Supplement 2). Fourteen patients with 140 tumors completed the trial, with 1 patient withdrawing (Figure 1B and C) because of shift-working patterns preventing compliance with the treatment application. Baseline data for this patient are included in accord with the statistical analysis plan, which has an intent-to-treat analysis specification. Skin adverse events were minor. Itch, which was transient, was reported in 2 actively treated tumors. One additional tumor underwent ulceration, which may have been related to active treatment. Patient compliance with treatment was excellent, with 98.8% of protocol treatment delivered. Treatment acceptability was positive, with 10 of 14 patients reporting that they would use the ointment as a treatment if it was effective.

**Figure 3.**
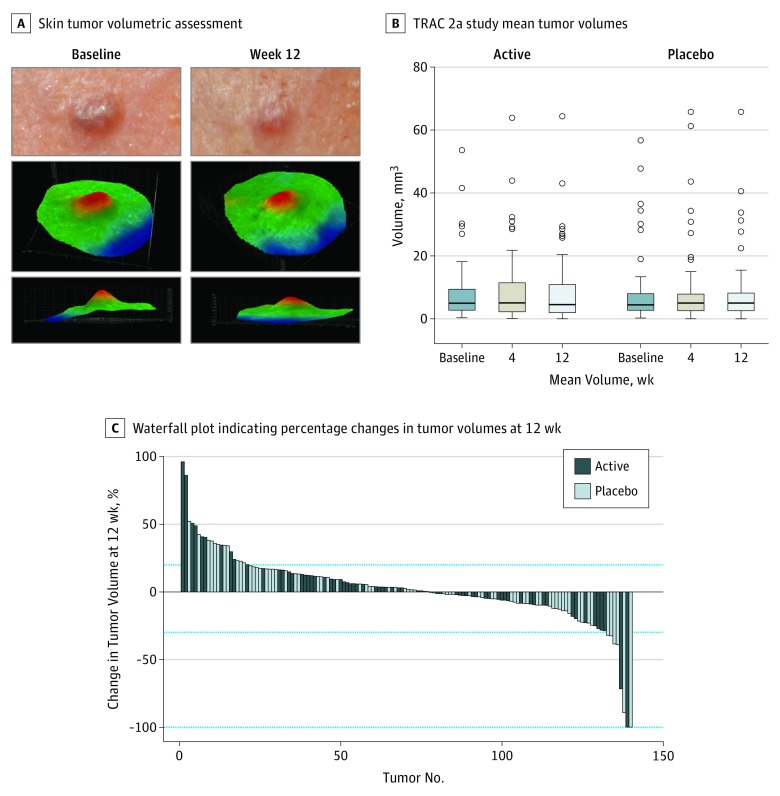
Tumor Volume Changes in Phase 2a A, Images taken at baseline and at week 12 of a shrinking tumor are shown, with 3-dimensional reconstructed surfaces of stereoscopic images illustrated from aerial and lateral perspectives. B, The median volumes of all tumors at baseline, 4 weeks, and 12 weeks in the active and placebo arms are shown in a Tukey plot. The upper error bar indicates the limit of values within quartile 3 plus 1.5 × (interquartile range), and the lower error bar indicates the limit of values within quartile 1 minus 1.5 × (interquartile range). C, A waterfall plot demonstrates changes seen in tumor volume expressed as a percentage change compared with baseline. TRAC indicates Tropomyosin Receptor Antagonism in Cylindromatosis.

In phase 2a, response to treatment was classified according to the World Health Organization–Response Evaluation Criteria in Solid Tumours (WHO-RECIST) criteria (http://recist.eortc.org/) as either a complete response or a partial response. Two tumors treated with pegcantratinib and 6 tumors treated with placebo were classified as responders. The prespecified critical number of tumors (n = 12) required to obtain a response in the actively treated tumors according to the statistical design was not met (Table and Figure 3). Tumor growth and shrinkage were seen in both actively treated tumors and placebo-treated tumors, and there was no significant difference in the mean volume at 12 weeks (difference of means, 3%; 95% CI, −6% to 7%). A priori specified analysis of tumors at terminal hair-bearing skin sites, such as scalp and vellus hair–bearing sites (nonhairy), did not reveal any trends in reduction in these subsets. Smaller tumors did not appear to respond differently than larger tumors.

**Table.** Response of Tumors in Participants Recruited to Phase 2a

Variable	No. (%) [95% CI]	
	Active (n = 70)	Placebo (n = 70)
Total responders	2 (2.9)	6 (8.6)
Complete response	1 (1.4) [0.2-9.9]	1 (1.4) [0.2-9.9]
Partial response	1 (1.4) [0.2-9.9]	5 (7.1) [2.9-16.4]
Total nonresponders	68 (97.1)	64 (91.4)
Stable disease	58 (82.9) [72.9-90.1]	53 (75.7) [64.0-84.5]
Progressive disease	10 (14.3) [7.7-24.9]	11 (15.7) [8.8-26.5]

Pain was detected in 14 of 140 tumors in phase 2a. Similar pain patterns were noted in actively treated tumors and placebo-treated tumors, suggesting that it was unlikely to be the active treatment causing pain. Eight painful tumors received active treatment. In 4 of these tumors, pain increased or remained the same, and pain decreased in the other 4 tumors. Six painful tumors received placebo treatment. In 5 of these tumors, pain increased or remained the same, and pain decreased in 1 tumor.

This trial opportunistically captured data on quality of life in CCS, and this was carried out using 2 validated tools at baseline only. The EQ-5D revealed a low effect of this disease on patients’ quality of life (eTable 4 in Supplement 2). Notably, in the pain/discomfort dimension, 6 patients reported moderate pain, and 2 patients reported severe pain. The DLQI, an effect measure designed for skin conditions, disclosed a small to moderate effect on quality of life, with a median DLQI of 4 (interquartile range, 2-8) (eTable 5 in Supplement 2).

**Figure 4.**
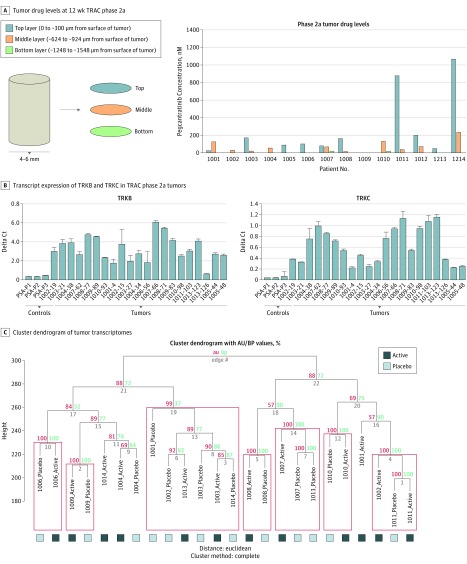
Drug Penetration Data and Expression of Drug Targets TRKB and TRKC From Biopsied Tumors at Week 12 in Phase 2a A, Skin biopsy specimens were snap frozen and serially sectioned, such that tissue was available at 3 levels in the tumor biopsy core, labeled top layer, middle layer, and bottom layer. Adjacent sections for histology were taken at the same time to confirm position within the core biopsy–included tumor cells. A liquid chromatography coupled to mass spectrometry (LC-MS) assay was used to measure levels of pegcantratinib at 3 levels within the tumors. B, Quantitative polymerase chain reaction was performed on RNA extracted from tissue sections taken adjacent to sections used for the top level of the drug assay. Increased expression of targets of pegcantratinib, TRKB and TRKC, in tumors compared with control skin samples is shown. The error bars indicate SEM. C, Unsupervised clustering analysis of transcriptomes generated from RNA extracted from tissue sections taken adjacent to sections used for the top level of the drug assay demonstrated frequent clustering by patient rather than by allocation to active or placebo treatments. AU Indicates approximately unbiased *P* value; BP, bootstrap probability value; and TRAC, Tropomyosin Receptor Antagonism in Cylindromatosis.

Access to skin biopsy specimens of tumor tissue treated in the trial allowed for in-depth molecular analyses. Histopathological analysis of 28 of 140 biopsied phase 2a tumors indicated that 25 (89.2%) were cylindroma (Figure 2C) and spiradenoma, with the remaining 3 (10.8%) being trichoepithelioma. Nerve stains of phase 1b tumor tissue demonstrated innervation of cylindroma and spiradenoma tumors (Figure 2D), a feature not previously demonstrated within these tumors.^16^ The drug penetration assay of 28 tumors was performed at 3 levels (Figure 4A). Pegcantratinib was demonstrated within multiple levels of tumor tissue in 12 of 14 pegcantratinib-treated tumors sampled. In certain tumors, drug was detected at all 3 levels, but in most tumors drug levels were only quantifiable in sections obtained from the top and middle levels. Corresponding placebo-treated tumors did not demonstrate the drug in these cases. The range of drug concentrations detected was from 13.61 to 1052 nM. Notably, 3-dimensional (3-D) culture of primary tumor cells demonstrated that 50% of cells are viable in concentrations of 19.2 μM (eFigure 1 in Supplement 2). The drug assay was also used at the end of the trial once unmasking was performed to check for compliance. In 1 of 14 individuals, we detected the drug on the side opposite to the allocated side. A sensitivity analysis was carried out to determine if excluding this individual would alter the outcome of the study, and that was found not to be the case. Overall, this supports that the trial design and delivery were robust, with a low rate of application error.

We assessed phase 2a tumors for expression of TRKs by carrying out RNA sequencing. Increased expression of *TRKB* and *TRKC* transcripts was seen in tumor tissue compared with normal skin, a finding that was validated using quantitative polymerase chain reaction (Figure 4B). Mutation analysis of RNaseq data did not show mutations in *TRKB* and *TRKC* kinase domains to account for inactivity or acquired resistance. Clustering analysis of transcriptomes (Figure 4C) demonstrated that most tumors clustered by patient rather than by allocation to active or placebo treatment. Downstream effects on proteins regulated by TRK signaling were assessed, namely, phosphorylated extracellular signal–regulated kinase (pERK) and B-cell lymphoma 2 (BCL2). pERK expression did not consistently reduce in the presence of active treatment (eFigure 2A in Supplement 2). BCL2 expression levels were reduced only in some pegcantratinib-treated tumors compared with levels in placebo-treated tumors and were unchanged or raised in others. No trend was evident from the data (eFigure 2B in Supplement 2), suggesting limited abrogation of TRK signaling in CCS tumors with the concentration of pegcantratinib used in this trial.

## Discussion

We report the first randomized, placebo-controlled trial to date in a large CCS cohort using tumor transcriptomic–led drug targeting. The inclusion of placebo-treated tumors in phase 2a revealed that some early CCS tumors may reduce in size, underscoring the importance of our trial design. As an intervention, we used pegcantratinib, 0.5% (wt/wt), an existing kinase inhibitor with activity against TRKA, TRKB, and TRKC designed for topical use, with an excellent safety profile in humans. We used a novel method of interventional trial in inherited human skin tumors that allowed us to overcome the failure of *Cyld* transgenic mice to develop cylindromas^1^ and provided tumor biopsy tissue to study the molecular effects of the intervention. We demonstrated that the drug penetrated tumor tissue. Most important, CYLD defective tumor primary cell culture models on 3-D tissue culture scaffolds showed sensitivity to low micromolar levels of pegcantratinib, supporting our rationale of in vivo targeting in patients with CCS. Drug measurement in treated tumor tissue demonstrated that high nanomolar concentrations were achieved in tumor cells with the applied concentration of pegcantratinib, which may account for the lack of clinical response seen. The absence of consistent changes in pERK and BCL2 in the actively treated tumors is consistent with this. Our data provide a rationale for dose-escalation studies of pegcantratinib or emerging systemic TRK inhibitors that have increasing safety data in humans.^17,18^

A clinically relevant observation was that pain reduction was reported in some tumors. Spiradenomas are recognized as 1 of 8 painful skin tumors in dermatology,^19^ and 50% of patients with CCS have reported a painful tumor.^2^ In the larger tumors seen in phase 1b, the reduction in pain seen in 3 painful tumors prompted us to study the smaller lesions for pain in phase 2a. Phase 2a tumors were less frequently symptomatic, and pain was only reported in 14 lesions, limiting the interpretation of our findings. Given the recognized activity of pegcantratinib in reducing itch,^8^ it is plausible that TRK inhibition, specifically of TRKA, within nociceptive pain fibers may account for this finding. We demonstrate multiple nerve fibers in these tumors, and these nerve cells may represent a second target in addition to cylindroma keratinocytes, where TRK is expressed.

### Limitations

Our trial had some limitations. This work highlights a limitation of repurposing in rare disease, where safety data allow quicker translation to the clinic, but the therapeutic effect may not be equivalent across diseases at the same concentration.

## Conclusions

This study highlights a novel approach using targeted therapeutics based on transcriptomic profiling in the rare inherited skin disease CCS. We investigated if pegcantratinib ointment, developed for inflammatory dermatoses (eg, psoriasis), could inhibit and possibly shrink skin tumors in CCS and consequently minimize the need for surgery in this rare condition. The excellent safety profile observed during this period of intervention supports the rationale for future research using dose-escalation studies of higher concentrations of pegcantratinib. Furthermore, some patients reported pain reduction in selected tumors, and additional research is needed to understand this observation. Finally, the careful study of these rare tumors has provided new data on the rate of tumor growth and their effect on quality of life, knowledge that will support the design of future trials in CCS.

## Notes

Supplement 1. Trial Protocol

Supplement 2. Materials and Methods **eMethods. **Supplementary Materials and Methods **eTable 1.** Medical Conditions and Concomitant Medications in Phase 2a Participants **eTable 2.** Tumor Characteristics From Participants Recruited to Phase 2a **eTable 3.** Tumor Measurements of all Tumors at Specified Time Points in Phase 2a Recruited Tumors **eTable 4.** Impact of Disease on Participants in Phase 1b & 2a as Measured by EQ-5D **eTable 5.** Impact of Disease on Participants in Phase 1b & 2a as Measured by DLQI **eTable 6.** Taqman Probes Used for Quantitative PCR **eFigure 1.** Primary Cultures **eFigure 2.** Protein Expression
