# Supplementary material for: Targeting Tropomyosin Receptor Kinase in Cutaneous CYLD Defective Tumors With Pegcantratinib: The TRAC Randomized Clinical Trial
Source: JAMA Dermatol. 2018 Jun 27;154(8):913–21. doi: 10.1001/jamadermatol.2018.1610 (PMC6128505; doi:10.1001/jamadermatol.2018.1610)
Supplement: Supplement 1. — Trial Protocol [file jamadermatol-154-913-s001.pdf]

An early phase trial of topical tropomyosin kinase (TRK) inhibitor as a treatment for inherited CYLD defective skin tumours

Short Title/Acronym: TRAC

EudraCT Number: 2014-001342-21

ISRCTN: 75715723

REC Reference: 14/NE/1080

R&D CSP Reference: 151395

Sponsor name and contact address:

Newcastle Upon Tyne Hospitals NHS Foundation Trust,
Freeman Hospital,
Freeman Road, High Heaton,
Newcastle-upon-Tyne, Tyne and Wear,
NE7 7DN

NUTH NHS Trust ref no: 6840

- Protocol Version & Date: V3.0, 16th May 2016
- Funder(s) references:
  Welcome Trust Ref: WT100935/Z/13/Z
  Department of Health Ref: HICF-R7-395

Contents

2. Glossary of Abbreviations 5
3. Responsibilities 7
4. Protocol Summary 9
 4.1 Cohort 1 10
 4.2 Cohort 2 10
5. Background 12
6. Objectives 15
 6.1 Cohort 1 15
 6.2 Cohort 2 15
7. Trial Design 16
 7.1 Cohort 1 summary 16
 7.2 Cohort 2 summary 17
8. Population 18
 8.1 Cohort 1 Inclusion criteria: 19
 8.2 Cohort 1 Exclusion criteria: 19
 8.3 Cohort 2 Inclusion criteria: 19
 8.4 Cohort 2 Exclusion criteria: 20
9. Screening, Recruitment and Consent 21
 9.1 Identification and screening of participants 21
 9.2 Recruitment procedures 21
 9.3 Consent procedures 21
 9.4 Acceptable Contraceptive Requirements 22
10. Trial medication 23
 10.1 General information 23
 10.2 Medication Blinding 23
 10.3 Cohort 1 23
 10.4 Cohort 2 23
 10.5 Drug Supplies 24
 10.6 Treatment Packaging and Labelling 24
 10.7 Drug Storage Conditions 24
 10.8 Drug Accountability 24
 10.9 Drug Administration 25
 10.10 Concomitant Medication 26
11. Randomisation (cohort 2 only) 26

12. Trial Blinding (Cohort 2 only) 27
13. Trial Data 28
13.1 Baseline Photography 28
13.2 Tumour volume assessment (Cohort 2) 28
13.3 Blood sampling 28
13.4 Schedule of Patient Assessments / Data Collection 28
13.5 Tumour Biopsies 31
13.6 Immunohistochemical Analyses and gene expression profiling 31
13.7 Schedule of Events: Cohort 1 33
13.8 Schedule of Events: Cohort 2 34
13.9 Data Handling and Record Keeping 36
14. Statistical Considerations 37
14.1 Statistical Definitions and Analysis 37
14.2 Number of Patients 38
14.3 Decision Criteria 38
15. Compliance and withdrawal 39
15.1 Assessment of compliance 39
15.2 Withdrawal of participants 39
16. Data monitoring, quality control and quality assurance 40
16.1 Data Monitoring 40
16.2 Discontinuation rules 41
17. Pharmacovigilance 42
17.1 Definitions 42
17.2 Expected adverse reactions: 43
17.3 Protocol Specifications 44
17.4 Recording & Reporting Serious Adverse Events or Reactions: 44
17.5 Pregnancies 46
18. Ethics & Regulatory Issues 47
19. Confidentiality 48
19.1 Safeguarding confidentiality 48
19.2 Long term data storage 48
20. Insurance and Finance 49
21. Trial Report / Publications 50
22. References 51
23. Appendices 52
23.1 Appendix A 52
23.2 Appendix B 53

Patient treatment questionnaire 53
23.3 Appendix C.. 54

2. Glossary of Abbreviations

**Abbreviation**	**Definition**
AD	Atopic dermatitis
ADR	Adverse Drug Reaction
AE/AR	Adverse Event/Adverse Reaction
BCC	Basal-cell carcinoma
BCL2	B-cell lymphoma 2
CIOMS	Council for International Organizations of Medical Sciences
CTA	Clinical Trial Agreement
CYLD	Cylindromatosis gene
CYLD	Cylindromatosis protein
DLQI	Dermatology Life Quality Index
DMC	Data Monitoring Committee
DNA	Deoxyribonucleic acid
DOH	Department of Health
e-CRF/CRF	electronic Case Report Form /Case Report Forms
ENT	Ear Nose and Throat
EQ5D	European Quality of Life-5 Dimensions
GCP	Good Clinical Practice
HICF	Health Innovation Challenge Fund
ICR	Institute of Cancer Research
IMP	Investigational Medicinal Product
LSE	Low systemic exposure
MAP	Mitogen-Activated Protein
MHRA	Medicines and Healthcare Products Regulatory Agency
NCTU	Newcastle Clinical Trials Unit

NuTH	The Newcastle upon Tyne Hospitals NHS Foundation Trust
PCI	Packaging Coordinators Inc
PIS	Patient Information Sheet
PTCH	Patched
QoL	Quality of Life
QPCR	Quantitative polymerase chain reaction
REC	Research Ethics Committee
RECIST	Response Evaluation Criteria In Solid Tumours
RNA	Ribonucleic acid
SAE/SAR	Serious Adverse Event/ Serious Adverse Reaction
SOP	Standard Operating Procedure
SUSAR	Suspected Unexpected Serious Adverse Reaction
TMG	Trial Management Group
TRK	Tropomyosin Related Kinase
TRKB	Tropomyosin Related Kinase B
TRKC	Tropomyosin Related Kinase C
TSC	Trial Steering Committee
WHO	World Health Organisation
WT	Wellcome Trust

3. Responsibilities

Sponsor: Newcastle Upon Tyne Hospitals will act as the sponsor for this trial.

Funder: Wellcome Trust HICF are funding this trial.

Trial Management: A Trial Management Group (TMG) will be appointed and will be responsible for overseeing the progress of the trial. The day-to-day management of the trial will be co-ordinated by the Newcastle Clinical Trials Unit (NCTU).

Chief Investigator: This is a single-centre trial and the Chief Investigator will have overall responsibility for the conduct of the trial at this site.

Trial Management:

The following functions falling under the responsibility of the sponsor will be delegated to Dr Neil Rajan, Chief Investigator:

- Authorisation and Ethics Committee Opinion (including CTA request, research ethics committee opinion, notification of protocol amendments and end of trial, site specific assessment & local approval)
- Good Clinical Practice and Trial Conduct (including GCP arrangements, management of IMP, data monitoring, emergency & safety procedures)
- Pharmacovigilance (including defining & recording adverse events/reactions, reporting SUSARs, notifying investigators of SUSARs, ensuring SAEs are reviewed by an appropriate committee for safety monitoring, annual listings & safety report).
- Administration of funding for the trial

Trial conduct at site:

Investigator responsibilities:

- Trial conduct and the welfare of trial subjects
- Familiarity with the use of the investigational medicinal product as described in the product information, appropriate storage, and administration according to the protocol and drug accountability. Ensuring investigational medicinal product is not used for any purposes other than the conduct of the trial.
- Compliance with the protocol, documentation of any protocol deviations and reporting of all serious adverse events
- Screening and recruitment of subjects
- Ensuring all trial-related medical decisions are made by a qualified physician, who is an investigator or co-investigator for the trial.
- Provision of adequate medical care in the event of an adverse event
- Obtaining local approval and abiding by the policies of Research Governance
    - Assistance will be provided by Newcastle Clinical Trials Unit

- Compliance with the Principles of GCP, the Research Governance Framework for Health and Social Care, and any national legislation implementing the EU Clinical Trials Directive (2001/20/EC) and subsequent amendments.
- Ensuring that no participant is recruited into the trial until all relevant regulatory permissions and approvals have been obtained.
- Obtaining written informed consent from participants prior to any trial specific procedures.
- The Principal Investigator (PI) shall be qualified by education, training and experience to assume responsibility for the proper conduct of the trial. S/he shall provide a current signed & dated curriculum vitae as evidence for the Trial Master File.
- Ensuring Trial Site team members are appropriately qualified by education, training and experience to undertake the conduct of the trial.
- Availability for Investigator meetings, monitoring visits and in the case of an audit.
- Maintaining trial documentation and compliance with reporting requests
- Maintaining a site file, including copies of trial approval, list of subjects and their signed informed consent forms
- Documenting appropriate delegation of tasks to trial personnel e.g. Pharmacist, Research Nurse, Investigator(s)
- Ensuring data collected is accurate, timely & complete
- Providing updates on the progress of the trial
- Ensuring subject confidentiality is maintained during the project and archival period
- Ensuring archival of trial documentation for a minimum of 15 years following the end of the trial, unless local arrangements require a longer period

4. Protocol Summary

Short title:	TRAC (Tropomyosin Receptor kinase Antagonism in Cylindromatosis)
Protocol version:	3.0
Protocol date:	16th May 2016
Chief Investigator:	Dr Neil Rajan
Sponsor:	Newcastle upon Tyne Hospitals NHS Foundation Trust
Funder:	Wellcome Trust HICF and Department of Health

Trial Summary:

Patients with germline mutations in a tumour suppressor gene called *CYLD* develop multiple, disfiguring, hair follicle tumours on the head and neck. The prognosis is poor, with up to 1 in 4 mutation carriers requiring complete surgical removal of the scalp. There are no effective medical alternatives to treat this condition.

This trial aims to repurpose a topical treatment with the potential to inhibit and possibly prevent tumour growth. Supported by a group of highly motivated patients, this work is driven by experimental data derived from rare, CYLD defective tumours. Whole genome molecular profiling experiments led to the discovery of an attractive molecular target in these skin tumour cells, named TRK.

Creabilis have recently developed an ointment (CT327) containing a TRK inhibitor (mini-pegylated - K252a). CT327 is an attractive, potentially novel agent for this condition as it is applied topically and already has safety data from human Phase 2b trials for the treatment of psoriasis and atopic dermatitis. This trial is designed to determine if the solution of delivering a small molecule to an inherited skin tumour model may represent a safe and feasible treatment for early tumours in these patients.

Trial design:	Cohort 1: A Phase 1b Open Label Clinical Trial<br>Cohort 2: A Phase 2a Randomised double blind exploratory placebo controlled trial
Trial Intervention:	CT327

4.1 Cohort 1

Primary objective:	To determine the safety of CT327 application in CYLD mutation carriers
Primary Outcome:	Number of patients with severe treated skin site reactions as determined by Modified Draize score
Secondary Outcome:	a. Patient reported quality of life using patient reported QoL tools (EQ5D, DLQI)<br>b. Acceptability of treatment according to patient treatment questionnaire (Appendix B).<br>c. Adverse events within a planned 4 week treatment period<br>d. Compliance including reasons for non-compliance
Number of trial sites:	1
Trial population/size:	8

4.2 Cohort 2

Primary objective:	To investigate if CYLD defective tumours respond to CT327
Secondary objective:	To delineate the mechanism by which TRK inhibition exerts on tumour volume.
Primary outcome:	The proportion of tumours responding to treatment by 12 weeks.
Secondary outcomes:	a. Change in tumour volume from baseline (pre-randomisation) to 12 weeks<br>b. Adverse events within a planned 12-week treatment period<br>c. Compliance including reasons for non-compliance<br>d. Confirmation of the definition of response (currently according to WHO RECIST criteria where response is defined as >30% reduction in tumour volume, this will be used as a benchmark).<br>e. Expression of targets of TRK signalling in tumour biopsies as determined by QPCR and immunohistochemistry.<br>f. Patient reported quality of life using patient reported QoL tools (EQ5D, DLQI)<br>g. Assessment of acceptability of trial treatment according to patient treatment questionnaire (Appendix B).<br>h. Patient reported pain using the Patient Pain Assessment Form at 0, 4 and 12 weeks.
Number of trial sites:	1

Trial population/size:	150 tumours with volume assessment at 12 weeks (anticipated in 15-20 patients)
Trial duration (Cohort 1+2):	30 months

5. Background

Clinical impact on patients with multiple CYLD defective tumours

Patients who inherit germline mutations in a tumour suppressor gene called *CYLD* develop multiple, disfiguring, hair follicle tumours on the head and neck. The prognosis is poor, with up to 1 in 4 mutation carriers requiring complete scalp excision typically at the age of 55.

These patients also have numerous tumours on the trunk that warrant surgical excision due to pain, ulceration or necrosis. Tumours have a predilection to form on the external ear and in the ear canal, resulting in conductive deafness as well as on genital skin, resulting in sexual dysfunction. These patients require repeated lifelong surgery to control tumour burden. There are no effective medical alternatives to treat this orphan disease, which is thought to affect approximately 1 in 100000 of the UK population. This trial aims to examine the potential of a repurposed topical treatment to inhibit and possibly prevent, tumour growth.

The impact of the disfiguring appearance on the quality of life of patients with this condition and the recurrent surgical treatments further emphasises the importance of this work. Patients find surgical interventions painful and time consuming, and may have limited function and ability to work in the weeks following surgery. This scenario, and its impact on NHS resources, could be revolutionised by an ointment that *CYLD* mutation carriers could apply to early tumours when they first develop. This may inhibit tumour growth and reduce the number of surgical interventions required. As these tumours often develop on the head and neck, the reduction in disfiguring tumours and surgery would have a positive effect on patient quality of life. Furthermore, the reduced referral for specialist interventions such as surgery and lasers would free up these precious resources as well as reduce the patient pathway time.

Current management of CYLD defective tumours

Currently, these tumours are excised by specialists in dermatology and plastic surgery, with patients requiring multiple procedures over the course of a lifetime. Some procedures such as laser resurfacing represent a costly and scarce resource. Extensive surgical procedures such as scalp excision and skin grafting may warrant a general anaesthetic and an inpatient stay. Patients with *CYLD* mutations have complex care needs, warranting the input of different specialists in their management.

There are <u>no effective medical alternatives</u> to treat this condition, and the TRK inhibitor, CT327, may represent a first in class agent for the management of this condition if shown to be safe and effective in this trial.

Patients with *CYLD* mutations have expressed that non-surgical alternatives would be an attractive way to manage their tumours. The delivery of CT327 as an ointment allows for high levels of the active ingredient to be delivered to the skin tumour cells, whilst avoiding the systemic side effects associated with oral formulations. Early treatment by informed patients would avoid specialist referrals and would confer a sense of control over this disfiguring disease to these patients. The reduction of surgical procedures on aesthetically important sites on the head and neck would improve patient's quality of life. Furthermore this would result in cost savings as the routine management of these tumours involves expensive interventions.

This early phase trial aims to evaluate an innovative approach towards managing the multiple tumours that arise in *CYLD* mutation carriers. This approach involves the use of an ointment containing a specific inhibitor (CT327) that targets a cell survival signal (TRK) present in the tumour cells in these patients. This targeting could lead to the inhibition of tumour growth and resolution of existing tumours. If proved effective by means of a future definitive trial, this treatment could lead to an improvement in quality of patient care and improved cost efficiency, which are key NHS priorities.

Scientific discovery of TRK as a therapeutic target in CYLD defective tumours

This trial is the culmination of a collaborative effort between researchers at Newcastle University and Prof Ashworth at the ICR. We searched for targetable kinases in inherited CYLD defective tumours using an unbiased approach, characterising whole genome DNA and RNA expression changes in fresh, snap-frozen tumours compared to adjacent, unaffected skin. This led to two key discoveries (Rajan, Ashworth *et al. Oncogene* 2011). Firstly, the genetic changes in these tumours were very consistent, with restriction to loss of *CYLD* being the only detectable change seen. This homogeneity was exciting as this implied that a targetable kinase discovered on this genetic background would be seen in the majority of tumours.

Secondly, we discovered overexpression of TRK selectively in the tumour cells . These tumour cells overexpressed two TRKs, TRKB and TRKC, in almost all tumours examined . The mechanism by which loss of functional CYLD results in perturbation of TRK homeostasis is not fully understood. TRK signalling has been shown to confer a survival advantage to tumour cells by increasing resistance to apoptosis and cell proliferation (Thiele, Z li et al. 2009; Jin, Kim et al. 2010; Ivanov, Panaccione et al. 2012). TRK has been increasingly recognised to be an oncogenic kinase that is overexpressed in several malignancies, including leukaemia and breast cancer. Our work also demonstrated the expression of TRK in common skin cancers such as BCC. The existence of preclinical TRK inhibitors allowed the testing of the utility of targeting TRK in CYLD defective cells. Transgenic mice that have been developed to explore the role of CYLD function fail to develop cutaneous tumours that recapitulate the human phenotype. Again, the ability to access fresh human tumours in Newcastle, allowed for the development of a CYLD defective tumour primary cell culture model on three dimensional tissue culture scaffolds. We demonstrated that this near-patient tumour model was highly sensitive to nanomolar levels of TRK inhibition. TRK inhibitors have so far been only available in oral formulations, but the advent of CT327, has made validation in a patient relevant model possible with minimal risk of systemic adverse effects.

CT327 is a TRK inhibitor that is already in use in human trials and can be repurposed

Our partnership with Creabilis will allow us to gain access to the first TRK inhibitor developed for topical use. Their proprietary molecule (CT327) uses LSE (Low systemic exposure) technology (a mini-pegylation approach) that generates molecules with physicochemical and pharmacological properties that make them suitable for topical use. TRK inhibition is a potential treatment in common inflammatory skin conditions, including psoriasis and eczema and Creabilis are currently exploring the drug in these areas, in Phase 2b studies. Safety data from studies completed to-date, where CT327 has been applied topically, at concentrations of up to 0.5% w/w for up to 8 weeks, in 36 healthy volunteers and 188 patients with psoriasis or eczema have shown that CT327 is well tolerated with a low incidence of adverse events and application site reactions. Pharmacokinetic samples taken in these studies confirm the absence of CT327 in the systemic circulation (assay detection level 5ng/ml) which is consistent with animal data and supportive of the theory that absorption of CT327 via skin is low following topical application.

Phase 2b trials in patients with psoriasis and atopic dermatitis are currently ongoing. No further refinement of the formulation would be needed to use this product in *CYLD* mutation carriers.

Proof of principle that inhibition of a key signalling pathway can restrain skin tumour growth has recently been described in another inherited skin tumour condition, Gorlin's syndrome. In this condition, the tumour phenotype conferred by germline mutations in a tumour suppressor gene *PTCH* are inhibited by a small molecule inhibitor targeting Hedgehog signalling, resulting in a reduction in tumour volume and the number of surgical procedures these patients needed (Tang *et al*., 2012).

The prospect of being able to inhibit TRK focally in the skin using an ointment vehicle for drug delivery, with the absence of the systemic side effects seen with other TRK inhibitors (Shabbir and Stuart 2010), is an attractive option, and is detailed in the trial design below.

The TRK trial is made up of 2 cohorts of patients with *CYLD* mutations. Cohort 1 is a phase 1b trial which aims to determine the safety profile of CT327 in 8 patients and will be followed by cohort 2, a phase 2a randomised, double blind, single-site trial to investigate the response of tumour if treated with CT327 .

6. Objectives

This is a non-commercial trial to determine the safety and preliminary efficacy of CT327 in patients with inherited CYLD defective skin tumours.

The trial is split into two phases namely a phase 1b which aims to determine the safety profile of CT327, and a phase 2a that will investigate if CYLD defective tumours respond to CT327.

6.1 Cohort 1

Primary objective:

To determine the safety of CT327 application in CYLD mutation carriers

A phase 1b open label clinical trial will be conducted to determine the safety of applying CT327 for four weeks to a single tumour in a CYLD mutation carrier that is scheduled for a routine lesion excision in 8 patients (detailed in section 8.1. below). The treated site will be clinically assessed using a modified version of the Draize score test for signs of local site reaction (See Appendix A).This is a measure of skin inflammation. The lack of any unexpected trial related adverse site reactions, as indicated by a modified Draize score of 3 or below, over 4 weeks of treatment in 5 out of 8 or more patients treated will allow progress to begin recruitment of patients into cohort 2.

6.2 Cohort 2

Primary Objective:

To investigate if CYLD defective tumours respond to CT327.

Cohort 2 is a phase 2a randomised double blind single site trial which aims to investigate if CT327 reduces tumour volume. This will be conducted in 15-20 patients over a 12 week period (detailed in section 8.2. below)

Secondary Objectives:

To delineate the mechanism by which TRK inhibition exerts on tumour volume.

In each patient we will excise two tumours, one treated with CT327 and the other treated with placebo. The placebo and CT327 treated samples will undergo gene expression profiling to delineate the mechanism by which TRK inhibition exerts an effect on tumour volume. Tumours will be assessed for changes following TRK inhibition using RNA sequencing to obtain changes in transcripts between placebo and CT327 treated tumours. This will give an insight into the effects of TRK inhibition in a CYLD defective tumour model, which will inform the understanding of the mechanism of this treatment in skin tumours.

A measure of the type and intensity of pain will be assessed in any of the selected tumours that the patient experiences pain in. Pain prior to the start of treatment and during the treatment period will be recorded.

7. Trial Design

7.1 Cohort 1 summary

This is a phase 1b open label clinical trial to determine the safety of applying CT327 to a pre-selected single tumour in a CYLD mutation carrier that is scheduled for routine excision .

Cohort 1 will determine the safety of targeting TRK in this patient population. A preliminary group of 8 patients (who are CYLD carriers) will receive daily doses (using a standardised spatula) of the topical TRK inhibitor CT327 (0.5%w/v) for 4 weeks to a pre-selected single tumour once a day.

As the gene dosage of *CYLD* in these patients is reduced in normal skin compared to healthy controls, this safety trial will determine safety in a *CYLD* mutation carrier population before looking at the efficacy of the drug in cohort 2.

Patients with a tumour that has been scheduled for excision will be eligible for inclusion in this cohort. Each patient will be required to apply CT327 ointment to the tumour only for the 4 week interval leading up to surgery following screening and enrolment into this trial. The treated site will be assessed by a clinician for signs of local site reaction. A patient treatment questionnaire (Appendix B) will be used to assess the patients' acceptability of the treatment.

Patients will also be asked to complete an EQ5D and DLQI before commencing treatment, in order to assess the impact of multiple CYLD defective tumours on their quality of life and to assess the patients' perception of the disease. This data has not been captured in this patient population previously. If the trial is successful and the treatment developed in future clinical trials this data could be looked at as a longitudinal measure.

Tumours excised will undergo routine analysis and some of the sample will be snap frozen and stored. This will be used for further mechanistic studies and stored for potential future analysis.

8.1.1 Cohort 1: Outcome measures

Primary Outcome Measure:	Number of severe treated skin site reactions defined as a Modified Draize score of 4 or above after 4 weeks of treatment (Appendix A)
Secondary Outcome Measures:	a. Patient reported QoL tools (EQ5D, DLQI)
	b. Patient acceptability of trial treatment according to patient treatment questionnaire (Appendix B).
	c. Adverse events within a planned 4 week treatment period
	d. Compliance as reported in the patient diary, including reasons for non-compliance

The decision to progress from cohort 1 to cohort 2 will be based on the number of severe skin reactions reported and on recommendations from the trial DMC.

8.1.2 Definition of end of Cohort 1 trial:

The end of cohort 1 will be once we have obtained the data on the last remaining cohort 1 patient after 4 weeks of treatment.

7.2 Cohort 2 summary

This is a phase 2a randomised, double-blind, single site, placebo controlled trial to investigate if CYLD defective tumours respond to CT327.

150 tumours will be needed to complete the trial, and recruitment will continue until we have the 12 week assessment for 150 tumours. We anticipate 15-20 patients may need to be recruited to the trial to achieve this.

Patients will typically have 8 or 10 eligible tumours each (see section 9.3 for cohort 2 inclusion criteria below).

Patients will be allocated both active and placebo treatments to be applied randomly to tumours on their left or right side (Group1: L=active; R=placebo, Group 2: L=placebo; R=active).

Four or five tumours on each side will be identified at baseline for measurements during the 12-week treatment period.

Photographs will be taken and the tumours numbered clearly and offered to the patients, who can refer to the tumour maps. They will be shown how to apply treatments using disposable finger tip applicators to prevent crossover and given a detailed instruction sheet for reference.

Volume measurements will be taken at baseline, week 4 and 12 at scheduled visits. All tumours will be assessed for tumour volume using the Life-Viz stereoscopic imaging system by a blinded assessor (Skvara, Kalthoff *et al.* 2011).

Patients will record application of treatment in a patient diary (See Appendix C).

Patients will also record if any of the selected tumours have any associated pain, this will be captured using the Patient Pain Assessment Form, which will be completed at baseline and repeated at 4 and 12 weeks.

After 12 weeks one tumour that is treated with active treatment and one tumour that has been treated with placebo will be biopsied to assess histological and immunohistochemical changes.

8.2.1 Cohort 2: Outcome measures:

Primary outcome measure:	The proportion of tumours responding to treatment by 12 weeks in both actively treated lesions and placebo treated lesions.
Secondary outcome measures:	a. Change in tumour volume from baseline (pre-randomisation) to 12 weeks assessed by a tumour volume measuring device, (Skvara *et al.*)
	b. Adverse events within a planned 12 week treatment period

c. Compliance as reported in the patient diary, including reasons for non-compliance

d. Confirmation of the definition of response (currently according to WHO RECIST criteria where response is defined as >30% reduction in tumour volume, this will be used as a benchmark).

e. Expression of targets of TRK signalling in tumour biopsies as determined by QPCR and immunohistochemistry.

f. Patient reported QoL tools (EQ5D, DLQI)

g. Assessment of acceptability of trial treatment according to patient treatment questionnaire (Appendix B).

h. Patient reported pain using the Patient Pain Assessment Form at 0, 4 and 12 weeks.

8.2.3 Definition of end of Cohort 2 trial:

The end of cohort 2 will be when the cohort 2 patient who brings the number of assessed tumours to 150 has had their 12 week assessment.

Three months (12 weeks) has been selected as the trial end point following discussion with 5 affected patients. It is in keeping with most topical treatments for skin growths, which are typically given for 1-3 months. It is expected that longer periods may have a negative effect on trial adherence.

8. Population

An overview of the tumours seen in these patients, the natural history and the distribution is relevant to the design and outcome measurement in this trial. Key points include:

1) *CYLD* mutation carriers develop multiple CYLD defective tumours, typically from the onset of puberty. These tumours (principally cylindromas, spiradenomas and trichoepitheliomas) are recognised to affect the head and neck as well as the torso and pubic skin.

2) We have studied the distribution of these tumours and they can be varied in size and location. Whilst penetrance is similar in males and females, severity appears to be increased in females. The tumour maps indicate the tumour locations documented in males and females in a study of 2 large Newcastle pedigrees (Rajan, Langtry *et al*. 2009).

These 2 pedigrees and a further 7 are well known to the Institute of Genetic Medicine and the department of dermatology in Newcastle. They are a highly motivated group of patients

and they have been central to the discovery of mutated *CYLD* in these patients' DNA led locally by Sir Prof John Burn (Bignell, Warren *et al*. 2000)

Adult patients will be recruited from genotyped pedigrees with known *CYLD* mutations, or if they have a clinical phenotype compatible with this diagnosis, namely multiple cylindromas, spiradenomas or trichoepitheliomas. The patients recruited in cohort 1 will be requesting and deemed suitable for surgery for tumour removal. These will typically be larger tumours that are painful or causing dysfunction, and the distinction from smaller lesions that are to be recruited in Cohort 2 is reflected in the respective inclusion criteria below.

8.1 Cohort 1 Inclusion criteria:

- Males and females age 18 years and older
- Patients from genotyped pedigrees with known *CYLD* mutations; or if they have a clinical phenotype compatible with this diagnosis
- Patients that are suitable for the trial will have at least one eligible tumour
- The eligible tumour will be scheduled for removal >4 weeks from consent
- The eligible tumour must be no more than 3cm in size
- For women of childbearing age: a negative pregnancy test is required prior to study entry, and on completion of trial treatment. The patient must be using an adequate contraception method (as defined in section 10.4) and agree to continue using this throughout the trial and for at least 2 weeks after stopping trial treatment
- Sexually active men must agree to use barrier forms of contraception
- The recruiting clinician must be confident that the patient understands the consent process and has the capacity and willingness to provide fully informed consent for participation in the trial

8.2 Cohort 1 Exclusion criteria:

- Patients aged <18 years
- Patients without CYLD defective tumours
- CYLD defective tumours which are ulcerated (these tumours will be managed according to standard practice of care)
- The eligible tumour is due to be removed <4 weeks from consent
- Pregnancy or lactation
- Women of childbearing age and sexually active men whom do not wish to use contraception whilst on the study
- Severe incapacity of higher function such that fully informed consent cannot be achieved, to be determined by clinical judgement
- Use of any other topically administered treatments at the treatment site

8.3 Cohort 2 Inclusion criteria:

- Males and females age 18 years and older
- For women of child bearing age: a negative pregnancy test is required prior to study entry, and on completion of trial treatment. The patient must be using an adequate contraception method (as defined in section 10.4) and agree to continue using this throughout the trial and for at least 2 weeks after stopping the trail medication
- Sexually active men must agree to use barrier forms of contraception
- Patients from genotyped pedigrees with known *CYLD* mutations, or if they have a clinical phenotype compatible with this diagnosis

- Patients will optimally have 8-10 eligible tumours
- Eligible tumours will be ideally less than 1 cm in diameter and no more than 2 cm in diameter at the base
- Eligible tumours must be spaced at least 1 cm apart from other eligible tumours to avoid cross-contamination
- The recruiting clinician must be confident that the patient understands the consent process and has the capacity and willingness to provide fully informed consent for participation in the trial
- Patients who have completed cohort 1without adverse reaction and after completing a minimum 2 week treatment free washout period

8.4 Cohort 2 Exclusion criteria:

- Patients aged <18 years
- Patients without multiple CYLD defective tumours
- Pregnancy or lactation
- Women of childbearing age and sexually active men whom do not wish to use contraception whilst on the study
- CYLD defective tumours which are ulcerated or have recently changed (these tumours will be managed according to standard practice of care)
- Severe incapacity of higher function such that fully informed consent cannot be achieved, to be determined by clinical judgement
- Significant concurrent illness
- Patients who developed an adverse reaction to CT327 in cohort 1 (score of 4 or above on the modified Draize score)
- Patients who have taken part in cohort 1and not completed a minimum 2 week treatment free washout period
- Large tumours >2cm base diameter will not be eligible
- Any tumour within 10cm of an excision scar of a cohort 1treated site will not be eligible
- Use of any other topically administered treatments at the treatment site

9. Screening, Recruitment and Consent

9.1 Identification and screening of participants

This is an exploratory trial to investigate the safety and efficacy of the TRK inhibitor CT327 on reducing the tumour size in patients with *CYLD* mutations. *CYLD* mutation carrier patients known to the clinical genetics and dermatology department in Newcastle will be reviewed by the clinical team to verify that they meet the trial inclusion criteria.

Eligibility screening logs will be completed by the investigator, or delegate, to document participants' fulfilment of the entry criteria for all patients considered for cohort 1 and cohort 2 and subsequently included or excluded. The eligibility screening logs will record the potential patient's initials, screening date, outcome of screening, reason patient ineligible/declined and consent date.

Screening logs will also record the number tumours each patient has for consideration for cohort 2.

In cohort 2 we will seek to recruit patients with 10 tumours in the first instance however we will reserve the option to recruit patients with 8 tumours each in case it becomes apparent that recruitment of an adequate number of patients with 10 tumours is problematic.

9.2 Recruitment procedures

Patients will be either approached during a routine outpatient appointment or written to and invited to attend a clinical appointment to discuss the trial.

Where patients are likely to be travelling long distances we will enclose a trial invitation letter and trial information sheet with their appointment letter. This will allow them more time to consider whether they are interested in taking part.

If a patient is happy to do so, it is feasible for screening, recruitment procedures and baseline visit to be completed over 24 hours and this may be necessary where patients are travelling long distances so that inconvenience to patients is minimised. Screening is also permitted to be a maximum of 7 days prior to randomisation in order to ensure that all screening data can be collected.

Female patients who are of child bearing age will be given urine based pregnancy tests prior to being randomised into either cohort 1 or cohort 2 of this trial.

9.3 Consent procedures

The clinical team members with delegated permission will explain the trial procedures to the patients and give them information about the trial treatments in cohort 1 or cohort 2 as appropriate (depending on which part of the trial is in progress). Prior to consent patients will also be given a trial Patient Information Sheet (PIS) to take away and read, they will be given opportunities to ask questions and as much time as they need (minimum 24 hours) to decide whether or not they wish to take part. Some patients, who are not under regular review, may have a PIS sent out in the post so they can have time to review the information at home and potentially consent at their next hospital visit for their convenience.

Those wishing to take part will provide written informed consent by signing and dating the trial consent form, which will be witnessed and dated by a member of the research team with documented, delegated responsibility to do so.

Only after written informed consent has been obtained will patients undergo any further trial specific screening (i.e. screening that cannot be done by simple review of medical notes-

such as pregnancy tests for example) and baseline clinical assessment, including skin examination.

The original signed consent form will be retained in the Investigator Site File, with a copy in the clinical notes and a copy provided to the participant. The participant will specifically consent to their GP being informed of their participation in the trial and a standard letter will be sent out. The Eligibility Screening Log will be updated to record the patient entering the trial.

The right to refuse to participate without giving reasons must be respected. Nevertheless, regardless of the reason, all refusals must be recorded in the Eligibility Screening Log.

Due to the small subject population, the PIS and consent form for the trial will be available only in English. Interpreters will be made available for all visits of patients who require them either for verbal translation or for deaf subjects wishing to take part in the trial, via local NHS arrangements. Qualified interpreters will be used to explain the consent form and information sheet, and great priority will be placed on finding the most direct communication. Consent will not be taken from participants unless the research team are confident they understand the information and can give fully informed consent.

The screening assessment (as per clinical practice) must be fully completed and eligibility confirmed by an investigator prior to enrolment (cohort 1) or randomisation (cohort 2). The trial drug can be started the same day as enrolment (cohort 1) or randomisation (cohort 2) if logistics permit.

9.4 Acceptable Contraceptive Requirements

Acceptable forms of effective contraception include:

1. Established use of oral, injected or implanted hormonal methods of contraception.
2. Placement of an intrauterine device (IUD) or intrauterine system (IUS).
3. Barrier methods of contraception: Condom or Occlusive cap (diaphragm or cervical/vault caps).
4. Male sterilisation (with the appropriate post-vasectomy documentation of the absence of sperm in the ejaculate). [For female subjects on the study, the vasectomised male partner should be the sole partner for that subject].
5. True abstinence: When this is in line with the preferred and usual lifestyle of the subject. [Periodic abstinence (e.g., calendar, ovulation, symptothermal, post-ovulation methods) and withdrawal are not acceptable methods of contraception].

10. Trial medication

10.1 General information

The TRK inhibitor, CT327, is a topical investigational medicinal product (IMP) currently being developed by Creabilis Limited for the treatment of pruritus and pain in dermatological conditions. This trial is the first trial to investigate the use of CT327 in patients with CYLD defective skin tumours, however the drug has been given to 36 healthy volunteers and 185 patients in previous clinical trials. Safety data can be found in the CT327 Investigator Brochure. Creabilis Ltd will supply study ointment (active and placebo) to the NuTH research pharmacy team for use exclusively in this trial. For reported side effects of CT327 please refer to Section 18 Pharmacovigilance.

Please refer to the Investigator Brochure for more detail.

10.2 Medication Blinding

10.3 Cohort 1

Cohort 1 is open label. Patheon UK Ltd (Kingfisher Drive, Covingham, Swindon, Wiltshire, UK) will be responsible for manufacture and packaging of the CT327 ointment. AndersonBrecon trading as Packaging Coordinators Inc (PCI) will be responsible for the labelling of the CT327 ointment and for sending a sufficient supply to NUTH pharmacy. The CI, or delegate, will notify the pharmacy when each patient is recruited to cohort 1 and complete the necessary prescription requests. NUTH pharmacy will then dispense the drug as required by the protocol.

10.4 Cohort 2

Cohort 2 is randomised and double blinded. Patheon UK Ltd will be responsible for manufacture and packaging of the CT327 and placebo ointment. AndersonBrecon trading as PCI will be responsible for the labelling of the supplies of CT327 and placebo ointment against the master randomisation list provided to them by the trial statistician. AndersonBrecon trading as PCI will supply sufficient CT327 and placebo ointment to NUTH pharmacy. The Chief Investigator and trial management team will be notified each time a patient is randomised by an automated email from the randomisation service backed up by notification via the research team (this is likely to be the person doing the randomisation, research nurse or trial manager depending on local arrangements). This information will enable the CI, or sub-investigator, to make the necessary prescription requests as per the randomisation allocation. NUTH pharmacy will then dispense the CT327 and placebo ointment to each patient as required.

The code for tumour treatment assignment will be kept locally by a third party who will not be responsible for patient care (in the hospital pharmacy). In the event of an emergency should the treatment assignment be needed, the clinical trials pharmacist at the Royal Victoria Infirmary (during normal working hours) or the on-call pharmacist (outside normal working hours) can be contacted to break the blinding (preferably with authorisation from the Chief Investigator) and the Chief Investigator immediately informed.

When the blinding code is broken the reason will be documented in the trial case report form (CRF) and in the patient's notes. See section 13 'Trial Blinding' for further information.

10.5 Drug Supplies

The study treatments are 0.5% w/w CT327 ointment or vehicle (placebo) for topical administration. The ointment contains butylated hydroxytoluene (BHT), benzyl alcohol, propylene glycol, white soft paraffin, white beeswax, stearyl alcohol and cholesterol. In addition the active ointment contains CT327. CT327 ointment and matching placebo ointment (vehicle) will be manufactured by Patheon UK Ltd (Kingfisher Drive, Covingham, Swindon, Wiltshire, UK). Patheon UK Ltd will package the CT327 ointment and matching placebo ointment (vehicle) in 20g amber glass jars. Labelling for ointments (for cohort 1 and cohort 2) will be performed by AndersonBrecon trading as PCI. The test materials will be identified by the lot, batch numbers, expiry date and certificate of analysis. CT327 ointment and vehicle will be supplied to NUTH pharmacy by AndersonBrecon trading as PCI and will arrive packaged and labelled as per the requirements of cohort 1 (unblinded CT327 ointment only) and cohort 2 (blinded CT327 ointment and vehicle according to the master randomisation list supplied by the trial statistician).

10.6 Treatment Packaging and Labelling

Patheon UK Ltd (Kingfisher Drive, Covingham, Swindon, Wiltshire, UK) will be responsible for treatment packaging. AndersonBrecon trading as PCI (Wye Valley Business Park, Brecon Street, Hay-on-Wye, Hereford, HR3 5PG, UK) will be responsible for treatment labelling. These products will be released to NuTH. AndersonBrecon trading as PCI will be responsible for labelling the active ointment for cohort 1(open label) and will be responsible for the labelling of the individual CT327 or vehicle ointment jars, with appropriate labels (cohort 1: CT327 0.5% w/w, cohort 2: CT327 0.5% w/w or placebo) according to the randomisation schedule for the trial.

10.7 Drug Storage Conditions

Study medication should be stored at ambient room temperature (15-25°C), in a controlled access area.

The ointments have a shelf life of 24 months at ambient temperature on the basis of ongoing 3 year ICH stability studies (as stated in investigators brochure).

10.8 Drug Accountability

AndersonBrecon trading as PCI will be responsible for certification of the finished IMP and will perform QP release. Study medication will be maintained under adequate security by appropriate site personnel (e.g. pharmacist) and in accordance with applicable regulatory requirements. Trial medication (including vehicle) remaining at the end of the trial will be returned to AndersonBrecon trading as PCI who can destroy and send destruction records to Creabilis.

Trial medication will be prescribed by a trial clinician, and dispensed to the patient or clinical staff according to local pharmacy policy. Patients in possession of their trial medication shall return all trial supplies in their original packaging (even if empty) to the research team who will pass it to the pharmacist at 4 weeks for cohort 1 and at 4 and 12 weeks for cohort 2. All returned, or unused, trial medication will be stored in pharmacy until the end of the trial, or until the trial manger has completed appropriate reconciliation in collaboration with pharmacy staff.

Documentation of prescribing, dispensing and return of trial medication shall be maintained for trial records.

Patient compliance with treatment will be monitored by means of patient diaries which will be reviewed by the trial team.

The investigator or designee must maintain adequate records of receipt and distribution of all trial medication, using appropriate accountability records.

10.9 Drug Administration

At the first trial visit, patients will have their tumours for drug administration identified; details for each cohort are stated in the inclusion/exclusion criteria (section 9). They will then be instructed on how to administer the trial medication and given patient instructions to take away for reference.

A new disposable finger stocking should be used for each separate administration and disposed of after use. The amount of drug will be measured using a provided spatula. The patient will also be instructed to wash the area with warm water before administration. The full tumour should be covered being careful to not administer treatment to any surrounding areas. The ointment should be spread evenly over the area. The ointment should be applied in the evening, 2 hours before bedtime.

11.7.1 Cohort 1

In cohort 1 active trial medication containing CT327 at 0.5%w/w will be provided as ointment in 20g glass jars. Patients will be provided with a spatula to help them to apply the correct amount of ointment.

The dose will be one application (2 standardised spatulas) in the evening to each tumour as directed at the first visit by the research nurse/doctor. Application will be recorded in a patient diary.

In cohort 1 participant's will treat their selected tumour with the active treatment for a 4 week period.

A telephone contact number will also be provided so participants can contact a member of the research team if required.

Any participant reporting significant side effects will be reviewed in clinic.

11.7.2 Cohort 2

In cohort 2 active and placebo trial medication will be provided at baseline and visit 4 to supply enough ointment for the 12 week period. Active medication will contain CT327 at 0.5%w/w. Each participant pack of active medication or placebo will be presented as a 20g glass jar.

The dose will be one application (1 standardised spatula) in the evening to each tumour as directed at the first visit by the research nurse/doctor. Patients will record application of treatment in a patient diary (Appendix C).

In cohort 2 all participants will be treating tumours on one half of the body with active CT327 and the other half of the body with placebo according to the randomisation allocation for a 12 week period. A telephone contact number will be provided so participants can contact a member of the research team if required.

Any participant reporting significant side effects will be reviewed in clinic.

A two day visit window is allowed for the second dispensing visit at week 4.

10.10 Concomitant Medication

For management of concomitant therapies, please refer to the Investigator Brochure. A complete listing of all concomitant medication received during the treatment phase must be recorded in the relevant CRF.

11. Randomisation (cohort 2 only)

A member of the trial team (as outlined in the delegation log) will re-check and confirm eligibility prior to randomisation. Eligibility can be confirmed by using information obtained from patient's notes and directly from the trial participants.

Randomisation will be at the per patient level randomising active and placebo treatments to left or right sided application. There will be no stratification at randomisation in this small early phase trial.

The statistics team will be responsible for preparing the allocation lists which will be uploaded to a secure web-based system by the database manager.

Randomisation will be administered centrally by the Newcastle Clinical Trials Unit internet-accessed secure web-based system which provides ease of operation, with in-built validation/plausibility checks at time of data entry.

The PI at site or an individual with delegate authority will access the web based randomisation system. Patient screening ID and initials will be entered into the web-based system. Patients will be allocated both active and placebo treatments to be applied randomly to left or right tumours (e.g. Group1: L=active; R=placebo, Group 2: L=placebo; R=active, where the terms L and R refer to the patient's left and right side). Patients allocated to a specific group (e.g. Group1: L=active; R=placebo), will remain in this same group when prescriptions are dispensed at baseline and week 4. Specific training will be given at site initiation.

The randomisation service website is accessed at: http://apps.ncl.ac.uk/random/

Queries about the randomisation system can also be addressed to:

nctu-enquiries@newcastle.ac.uk

Practicalities of the randomisation of tumours to each arm of the trial:

4 or 5 tumours matched for size will be selected on each side of the patient. Each tumour will be assigned a number to facilitate assessments and then each side of the patient will be allocated randomly to receive either active ointment or placebo ointment.

Photographs will be taken and the tumours numbered clearly so that the patient can refer to the tumour maps to ensure the correct tumour is being treated from the correct ointment jar.

Tumours will be marked with a 2mm dot using a permanent marker designed for use on the skin, which the patient will renew as necessary. Tumours within the hair will have a small amount of surrounding hair trimmed to allow the patient to locate the tumour by feel, and for volume assessments.

12. Trial Blinding (Cohort 2 only)

Patients and investigators will be blinded to the treatment allocation. Those responsible for tumour volume measurements, histology assessments and molecular analysis will also be blinded to treatment allocation. The Trials Management Group including Trial Manager will be blind to the treatment allocation. The trial statistician may provide confidential unblinded data to the closed session of the external independent data monitoring committee (excluding members of the Trials Management Group).

Following the unlikely event of a code break, patients' will continue to be followed up if they agree to do so. If the unblinding reveals that the cause was the placebo ointment, patients will be offered the continuation of the trial medication as per the randomisation if they wish. The analysis of the trial will reflect the unblinding, data both including and excluding the unblinded participants will be analysed to avoid bias.

Code breaks will not be routinely performed for all participants who complete trial treatment but all patients may be informed of their allocations once the trial analysis is complete on request and the data released to the chief investigator.

At the final visit, the integrity of the blind will be assessed by asking the participants: Which side was receiving active treatment? Why do you think this?" on the patient treatment questionnaire. The treatment assessor will be asked to record their answer on a separate CRF and prior to asking the participant to avoid bias.

13. Trial Data

13.1 Baseline Photography

Cohort 1: Baseline photographs of the selected tumour, scheduled for excision, will be taken for reference for the 8 patients in cohort 1.

Cohort 2: Photographs with clearly numbered tumours will be offered to the patients so they can refer to the tumour maps to ensure the correct tumour is being treated from the correct ointment jar and kept at site for reference. Tumours will be marked with a 2mm dot using a permanent marker designed for use on skin, which the patient will renew as necessary. Tumours within the hair will have a small amount of surrounding hair trimmed to allow the patient to locate the tumour by feel, and for the volume measurements.

13.2 Tumour volume assessment (Cohort 2)

All tumours will be assessed for tumour volume using the Life-Viz stereoscopic imaging system by a blinded assessor (Skvara, Kalthoff et al. 2011). This is a validated system that has been shown to be a highly sensitive and reproducible method to assess skin tumour volume assessments. The device (6a) will be used to measure the volume of individual tumours (6b). The region of interest, once defined, will be measured as a base area and a raised volume compared to surrounding skin (c; i-iv). This measurement of volume will be taken at 0, 4 and 12 weeks in the trial for each tumour.

13.3 Blood sampling

Blood samples will be processed and stored for potential future analysis as per the lab handling manual.

13.4 Schedule of Patient Assessments / Data Collection

14.4.1 Cohort 1

Visit 1 – Pre Screening/Baseline visit

- Written informed consent will be taken as described in the section 10.3 of this protocol.
- Inclusion criteria will be checked and patients will be assessed to ensure they have an appropriate tumour for the trial treatment, this tumour must be scheduled for routine excision and must not be ulcerated.
- Confirmation must be recorded in the CRF that trial inclusion criteria are fulfilled and no exclusion criteria apply.

Visit 2 – Screening/Baseline visit

The baseline visit will involve collection and retrospective collation of the following data:

- Medical history and demographics (age, gender, race)
- Tumour selected for trial treatment
- Baseline photography

- Pregnancy Test (urine), for women of child bearing potential only
- Patient reported measures: EQ5D and DLQI
- Concomitant Medication
- Confirmation must be recorded in the CRF that trial inclusion criteria are fulfilled and no exclusion criteria apply.
- Participants will be told how to apply the trial treatment and given a patient instruction sheet to take away for reference. They will also be reminded of the contact numbers stated in the patient information sheet and encouraged to call if they have any queries.
- Provide patient with Patient Diary.

Visit 3a – Week – 4 End of treatment

Visit 3 will take place at the end of treatment 4 weeks (day 28 +3days) after the entry to trial.

- Pregnancy Test (urine) , for women of child bearing potential only
- Patient treatment questionnaire
- Adverse Events
- Concomitant Medication
- Trial medication returned
- Collect completed Patient Diary

Visit 3b – Surgery

The surgery will take place at the Week 4 Visit once treatment assessment is complete

- Lesion excision

14.4.2 Cohort 2

Visit 1 – Pre Screening/Baseline visit

- Written informed consent will be taken as described in the section 10.3 of this protocol.
- Inclusion criteria will be checked and patients will have appropriate tumours selected for the trial treatment.
- Confirmation must be recorded in the CRF that trial inclusion criteria are fulfilled and no exclusion criteria apply.

Visit 2 – Screening/Baseline visit

Randomisation should occur after pregnancy test and checking of inclusion/exclusion criteria. Four or five tumours will be matched for size as closely as possible and each one assigned a number a facilitate assessments and then each side of the patient allocated randomly to receive either active or placebo ointment. Jar numbers will then be allocated according to the master randomisation list.

Once patient has been randomised the trial medication can be dispensed.

The baseline visit will involve collection and retrospective collation of the following data:

- Medical history and demographics (age, gender, race)
- Baseline photography (as described in section 14.1)
- Tumour volume assessment (as described in section 14.2)
- Tumour pain assessment (log sheet and a week 0 Pain Assessment Form to be completed for each selected tumour).
- Pregnancy Test (Urine), for women of child bearing potential only
- Patient reported measures: EQ5D and DLQI (except if patient was previously in cohort 1and a measurement exists)
- Concomitant Medication
- Patient provided with Patient Diary

Week 2 - Telephone call

- Patients will be contacted via telephone after 2 weeks to ask if they are managing the administration of the ointment and if they have any concerns or questions.

Visit 3 – Week 4- Treatment assessment

Visit 3 will take place at 4 weeks (+/- 3 days) after the randomisation date.

- Pregnancy Test (Urine) , for women of child bearing potential only
- Tumour volume assessment
- Tumour pain assessment (log sheet and a week 4 Pain Assessment Form to be completed for each selected tumour).
- Adverse Events
- Concomitant Medication
- Trial medication returned
- Trial medication dispensed (according to randomisation email at baseline)
- Patient Diary reviewed by Investigator or delegate

Week 6- Telephone call

- Patients will be contacted via telephone after 6 weeks to ask if they are still managing the administration of the ointment and if they have any concerns or questions.

Visit 4 –Week 12 - End of treatment

The Week 12 Visit will take place at 12 weeks (+/- 3 days) after the randomisation date.

- Blood sample to be processed and stored - plasma
- Pregnancy Test (urine), for women of child bearing potential only
- Tumour volume assessment
- Tumour pain assessment (log sheet and a week 12 Pain Assessment Form to be completed for each selected tumour).
- Adverse Events
- Patient treatment questionnaire
- Concomitant Medication
- Trial medication returned
- Tumours biopsied
- Collect completed Patient Diary

Telephone call (4 week follow up)

Participants will be contacted by telephone 4 weeks after completing the trial to ensure there have been no further significant adverse events during this period. They will be asked if they have experienced any treatment site reactions since stopping treatment, or any other side effects. If necessary an appointment will be made for the participant to have a follow up visit in clinic for assessment and management.

13.5 Tumour Biopsies

Cohort 1: At the end of treatment visit, week 4, the routinely excised tumour will undergo routine analysis and some of the sample will be snap frozen and stored. This sample will be stored with a view to developing a CT327 skin assay. Participants will have the option to consent to samples potentially being used in future studies relating to the title of this trial.

Cohort 2: At the end of treatment visit, week 12, one tumour that has been treated with active treatment and one tumour that has been treated with placebo will be biopsied to assess histological and immunohistochemical changes. A proportion of these samples will be stored with the potential to undergo CT327 analysis in the future.

13.6 Immunohistochemical Analyses and gene expression profiling

A secondary endpoint is histological and immunohistochemical changes, and the two biopsied samples from cohort 2 will be analysed to assess such changes.

These tumour samples will be assessed by a pathologist that is blinded to the treatment applied to the specimens. Immunostaining with phosphorylated forms of the MAP kinase pathway and BCL2 (an antiapoptotic protein) will be assessed to determine if TRK inhibition is leading to anticipated cell death via suppression of the expected downstream targets of

TRK. Tissue concentrations of CT 327 will also be assessed in a representative portion of samples.

To fulfil the secondary objective for cohort 2, delineate the mechanism by which TRK inhibition exerts an effect on tumour volume, the following procedures will be performed

Gene expression profiling of tumour samples to compare placebo and control treated samples. Tumours will be assessed for changes following TRK inhibition using RNA sequencing to obtain changes in transcripts between placebo and CT327 treated tumours. A total of 12 treated and 12 placebo tumours will be assessed, which we plan to obtain from the 15-20 recruited patients. This will give insight into the effects of TRK inhibition in a CYLD defective tumour model, which will inform the mechanistic understanding of this treatment in skin tumours.

13.7 Schedule of Events: Cohort 1

Time	Visit 1<br>Pre-Screening	Visit 2<br>Baseline visit<br>Confirmation of eligibility			Visit 3a<br>Week 4 EoT	Visit 3b<br>Surgery
	—	Week 0			28 days<br>(+3 days)	28 days<br>(+3 days)
Trial Discussed / PIS given[1]	X					
Informed Consent[2]		X				
Photography				X		
Medical History and demographics			X			
EQ5D			X			
DLQI			X			
Patient treatment questionnaire					X	
Pregnancy Test[3]				X	X	
Trial medication dispensed				X		
Trial medication returned					X	
Patient diary				X	X	
Lesion Excision						X
Adverse events					X	
Concomitant medications			X		X	
CRF completion				X	X	

[1] Patient information sheet can be post to avoid any unnecessary journey
[2] A minimum of 24hr for review of patient information sheet before patient can sign informed consent form
[3] Pregnancy test should be urine dipstick and for all women of childbearing potential

13.8 Schedule of Events: Cohort 2

Time	Visit 1<br>Pre-Screening	Visit 2<br>Baseline visit<br>Confirmation of eligibility & Randomisation			Telephone call	Visit 3<br>Week 4 Treatment Visit	Telephone call	Visit 4<br>Week 12 EoT	Telephone call
	Week -1	Week 0			Week 2	Post Week 4	Week 6	Post week 12	4 weeks post visit 4
Trial Discussed / PIS given[1]	X								
Informed Consent[2]		X							
Photography				X					
Medical History and demographics			X						
EQ5D[3]			X						
DLQI[3]			X						
Patient treatment questionnaire								X	
Compliance assessment					X		X		
Volume measurement				X		X		X	
Tumour Pain Assessment				X		X		X	
Pregnancy Test[4]			X			X		X	
Blood sample								X	
Trial medication dispensed				X		X			
Trial medication returned						X		X	
Patient Diary				X		X		X	

Lesions biopsied								X	
Adverse events						X		X	X
Concomitant medications			X			X		X	
CRF completion				X		X		X	X

[1] Patient information sheet can be post to avoid any unnecessary journey
[2] A minimum of 24hr for review of patient information sheet before patient can sign informed consent form
[3] QoL only to be completed if patient was not part of the cohort 1 trial.
[4]Pregnancy test should be urine dipstick and for all women of childbearing potential.

13.9 Data Handling and Record Keeping

The quality and retention of data that is collected as part of this trial is under the responsibility of the Chief Investigator, Dr Neil Rajan.
All trial data will be retained in accordance with the latest Directive on GCP (2005/28/EC) and local policy. Data will be handled, computerised and stored in accordance with the Data Protection Act 1998. Patients will be identified by a unique trial number. Data will be recorded by authorised site staff on electronic Case Report Forms (eCRFs) and stored on a secure validated MACRO database system managed by Newcastle Clinical Trials Unit. Caldicott approval will be sought at The Newcastle upon Tyne NHS Foundation Trust site to enable the collection of personal identifiable information for purposes of trial administration (including arranging patient assessments) and maintaining accurate screening records.

14. Statistical Considerations

14.1 Statistical Definitions and Analysis

All statistical analysis will follow a trial-specific predetermined statistical analysis plan (SAP), written and signed off prior to any statistical analysis of the trial data (excepting recruitment) and stored in the TMF.

2.1.1 Cohort 1

All patients will contribute to the analysis based on the total number recruited.

The primary outcome measure is the number of patients with severe or very severe treated skin site reactions as determined by modified Draize score (See Appendix A)

Patient perception of treatment is a secondary outcome measure as measured by the patient treatment questionnaire (See appendix B). Positive patient perception will be reported descriptively as a proportion of the number of patients recruited.

Statistical analysis for all outcome measures will be exploratory based on descriptive data. Continuous measurements (including QoL measures) will be summarised and reported as medians and ranges (inter-quartile ranges). Categorical data (including proportions of patients with positive patient perception and with adverse events) will be summarised and reported as percentages and confidence intervals (95%).

QoL tools (EQ5D, DLQ) will be scored according to scoring manuals and actual scores will reported descriptively.

2.1.2 Cohort 2

All tumours will contribute to the analysis based on the total number randomised (intention to treat population). A secondary analysis will be reported based on the number of tumours which have been treated for 12 weeks (per protocol population).

The primary outcome measure is the number of tumours responding to treatment by 12 weeks reported as a proportion of the number of tumours randomised in the actively and placebo groups separately.

According to WHO-RECIST criteria, a reduction in 30% volume would be sufficient to classify a ‘response’ to treatment in oncology. In this trial we will used this criteria as a bench mark when determining a response in this patient population. As such one of the outcomes will be to consider and confirm a definition of ‘response’ to treatment in this group of patients. Tumour volume will be measured on 8 - 10 predetermined identified tumours in each patient prior to randomisation (baseline) and measured again on the same tumours at 4 and 12 weeks. The change in volume for each tumour will be recorded and classified as either having a response to treatment or not.

As an early phase trial, statistical analysis will be exploratory (not based on hypothesis testing) and will be based on descriptive data presented by treatment group. Continuous measurements (including change in tumour volume from baseline) will be summarised and reported as medians and ranges (inter-quartile ranges). Categorical data (including proportions of tumours with adverse events and outcomes from the Pain Assessment Forms) will be summarised and reported as percentages and confidence intervals (95%). Since multiple tumours are sited in individual patients, a secondary analysis based on a multi-level

modelling approach will be undertaken to investigate the relationship between any change in volume and randomised treatment accounting for the multi-level nature of the tumour measurements.
QoL tools (EQ5D, DLQ) will be scored according to scoring manuals and actual scores will reported descriptively.

14.2 Number of Patients

Cohort 1

It is anticipated that there will be low numbers of treatment related adverse events in Cohort 1 and it is therefore both feasible and pragmatic to recruit 8 patients. It will be acceptable to move to cohort 2 as long as any unexpected treatment related adverse events are experienced in no more than 3 patients.

Cohort 2

It is anticipated that 75 tumours could be measured in each treatment arm (150 tumours in total). Tumours receiving placebo treatment are not expected to respond to treatment, set to be very small at 5% (p0). This is the first exploratory trial and as such the design parameters are provided as an exemplar of the size of errors that may be anticipated with these patient numbers.
Using Fleming A'Herns early phase methodology (Fleming 1982, Ahern, 2001) any response on the experimental treatment <5% (p0) would not indicate a treatment worthy of further investigation. 75 tumours recruited in the experimental arm would provide evidence of a minimum level of efficacy of approximately 15% (p1), indicating that a level of efficacy of >15% would warrant further investigation, with associated error levels of 3.4% type I error (alpha) and 10.8% type II error (beta). This level of efficacy seems clinically plausible and relevant given that there is no current treatment in use for these patients.
The justification to investigate CT327 further is based on observing a minimum number of responses, referred to as the critical number (specified in the Statistical Analysis Plan).
The trial will recruit an equal number of placebo-treated tumours to provide an unbiased benchmark.

14.3 Decision Criteria

Cohort 1

We anticipate recruiting 8 patients to cohort 1. It will be acceptable to move to cohort 2 as long as any unexpected treatment related adverse events are experienced in no more than 3 patients.

Cohort 2

The Fleming A'Hern design would indicate no further investigation is warranted if the observed number of clinical responses is less than the critical number (as specified in the Statistical Analysis Plan).

15. Compliance and withdrawal

15.1 Assessment of compliance

Where feasible, trial visits will coincide with routine clinical follow-up, to enhance the likelihood of good compliance. Visit windows of +/- 3 days should ensure visit attendance; non-attendance for trial visits will prompt follow-up by telephone.

Compliance with trial medication will be assessed by checking the patient diaries and weighing and recording the returned jar after each treatment visit and at the final visit. Trial drug accountability will be assessed and documented by local pharmacy. The clinical team will also perform a quick review of any returned trial medication at each trial visit to identify any obvious compliance concerns and address these immediately with the participant.

15.2 Withdrawal of participants

Trial drug must be discontinued if:

- The participant decides they no longer wish to continue
- Cessation of trial drug is recommended by the investigator
- Pregnancy
- Any significant medical condition (in the judgement of the site investigator)
- Localised reactions:

Any patient reporting treatment site reactions will be assessed in the clinic. A modified Draize score of 4 or above would result in discontinuation of the drug. In the event of such an adverse reaction, blinding may need to be broken to determine if the reaction is related to the application of the trial drug to the treated site. Once the blind is broken, the trial drug will need to be discontinued if it is found to be responsible for the adverse event.

- Systemic reactions:

Any other reported side effects/adverse events that the patient experiences during the trial will be assessed by the chief investigator. If clinically appropriate, the patient will be reviewed in clinic and a decision made regarding discontinuing the trial drug if it is feasible that this is related to the trial drug.

Participants have the right to completely withdraw from the trial at any time for any reason, and without giving a reason. The investigator also has the right to withdraw patients from the trial drug in the event of inter-current illness, adverse events, serious adverse events, suspected unexpected serious adverse reactions, protocol violations, cure, administrative reasons or other reasons. It is understood by all concerned that an excessive rate of withdrawals can render the trial uninterpretable; therefore, unnecessary withdrawal of patients should be avoided. Should a patient decide to withdraw from the trial, all efforts will be made to report the reason for withdrawal as thoroughly as possible. Should a patient withdraw from trial drug only, efforts will be made to continue to obtain follow-up data, with the permission of the patient.

Participants who wish to withdraw from trial medication will be asked to confirm whether they are still willing to provide the following.

- ‘end of trial data’ as per visit 3 (4 weeks) in cohort 1 or visit 4 (12 weeks) in cohort 2, at the point of withdrawal

- If participants agree to the above, they will be asked to complete a confirmation of withdrawal form to document their decision.

In cohort 1, participants who withdraw from trial drug prior to visit 3 will still be followed up and included in analysis, if they are happy to do so, but additional participants may be recruited provided withdrawal is not related to safety issues.

In cohort 2, participants who withdraw from trial drug will still be followed up and included in analysis, if they are happy to do so, but additional participants may be recruited and randomised as not to affect the recruitment target of 150 tumours and provided withdrawal is not related to safety issues.

16. Data monitoring, quality control and quality assurance

The trial may be prematurely discontinued on the basis of new safety information, or for other reasons given by the Data Monitoring Committee and/or Trial Steering Committee, Sponsor, regulatory authority or ethics committee concerned.
The trial will be managed through NCTU. The Chief Investigator Dr Neil Rajan will be responsible for the day-to-day trial conduct at site.
NCTU will provide day-to-day support for the site and provide training through Investigator meetings, site initiation visit and routine monitoring visits.
Quality control will be maintained through adherence to NCTU SOPs, trial protocol, Statistical Analysis Plan (SAP), the principles of GCP, research governance and clinical trial regulations.

An independent data monitoring committee (DMC) (2 physicians not connected to the trial, one statistician) will be convened to undertake independent review. The purpose of this committee will be to monitor safety in cohort 1 and recruitment, safety and outcomes in cohort 2. The DMC may request access to unblinded trial data. The DMC will meet at least 3 times; at the start, following completion of cohort 1 data collection and following completion of cohort 2 data completion. At the first meeting, DMC will agree meeting timelines and discuss stopping guidelines.

A Trial Steering Committee (TSC) will be established to provide overall supervision of the trial. The TSC will consist of independent chair, further independent clinician,, independent consumer representative, funder representative & Trial Management Group. The committee will meet before the start of the trial to approve the protocol and at least annually thereafter for the duration of the trial. The Chair of the TSC and the PI can request meetings more frequently if needed and if also requested by the DMC.

16.1 Data Monitoring

Monitoring of trial conduct and data collected will be performed by a combination of central review and site monitoring visits to ensure the trial is conducted in accordance with GCP. Trial site monitoring will be undertaken by NCTU. The main areas of focus will include consent, serious adverse events, essential documents in trial files and drug accountability & management.
Site monitoring will include:
- All original consent forms will be reviewed as part of the site file. The presence of a copy in the patient hospital notes will be confirmed for 100% participants

- All original consent forms will be compared against the trial participant identification lists
- All reported serious adverse events will be verified against treatment notes/medical records (source data verification)
- The presence of essential documents in the investigator site file and site files will be checked
- Source data verification of primary endpoint data and eligibility data for 100% of participants entered in the trial cohort 1 and 50 % of participants in trial cohort 2.
- Drug accountability and management will be checked

Central monitoring will include:

- All applications for trial authorisations and submissions of progress/safety reports will be reviewed for accuracy and completeness, prior to submission
- All documentation essential for trial initiation will be reviewed prior to site authorisation

All monitoring findings will be reported and followed up with the appropriate persons in a timely manner.
The trial may be subject to inspection and audit by Newcastle Upon Tyne Hospitals NHS Foundation Trust (NUTH) under their remit as sponsor, and other regulatory bodies to ensure adherence to GCP. The investigator(s) / institutions will permit trial-related monitoring, audits, REC review and regulatory inspection(s), providing direct assess to source data/documents.

16.2 Discontinuation rules

The trial may be prematurely discontinued on the basis of new safety information, or for other reasons given by the Data Monitoring Committee and/or Trial Steering Committee, Sponsor, regulatory authority or ethics committee concerned.

If the trial is prematurely discontinued, active participants will be informed and no further participant data will be collected.

17. Pharmacovigilance

17.1 Definitions

Adverse event (AE): Any untoward medical occurrence in a subject to whom a medicinal product has been administered, including occurrences which are not necessarily caused by or related to that product. An AE, therefore, does not necessarily have a causal relationship with the treatment. In this context, “treatment” includes all investigational agents (including comparative agents) administered during the course of the trial. Medical conditions/diseases present before starting trial treatment are only considered adverse events if they worsen after starting trial treatment.

Adverse Reaction (AR)/Adverse Drug Reaction (ADR): Any untoward and unintended responses to an Investigational Medicinal Product (IMP) which related to any dose administered to that subject. All AEs judged by either the reporting investigator or the sponsor as having reasonable causal relationship to a medicinal product qualify as adverse reactions. The expression “reasonable causal relationship” means to convey in general that there is evidence or argument to suggest a causal relationship.

Causality:

The assignment of the causality should be made by the investigator responsible for the care of the participant using the definitions in the table below. All adverse events judged as having a reasonable suspected causal relationship to the IMP(s) (i.e. definitely, probably or possibly related) are considered to be adverse reactions. If any doubt about the causality exists, the Chief Investigator should be informed. In the case of discrepant views on causality between the investigator and others, all parties will discuss the case. In the event that no agreement is made, the MHRA, main REC and other bodies will be informed of both points of view.

**Relationship**	**Description**
Unrelated	There is no evidence of any causal relationship
Unlikely	There is little evidence to suggest there is a causal relationship (e.g. the event did not occur within a reasonable time after administration of the trial medication). There is another reasonable explanation for the event (e.g. the participant’s clinical condition, other concomitant treatment).
Possible	There is some evidence to suggest a causal relationship (e.g. because the event occurs within a reasonable time after administration of the trial medication). However, the influence of other factors may have contributed to the event (e.g. the participant’s clinical condition, other concomitant treatments).
Probable	There is evidence to suggest a causal relationship and the influence of other factors is unlikely.
Definitely	There is clear evidence to suggest a causal relationship and other possible contributing factors can be ruled out.
Not assessable	There is insufficient or incomplete evidence to make a clinical judgement of the causal relationship.

Unexpected Adverse Reaction: An adverse reaction the nature and severity of which is not consistent with the information about the medicinal product in question set out:- (a) In the case of a product with a marketing authorisation, in the Summary of Product Characteristics for that product; (b) in the case of any other investigational medicinal product, in the Investigator's Brochure relating to the trial in question.

Serious Adverse Event (SAE) or Serious Adverse Reaction (SAR): an adverse event, adverse reaction or unexpected adverse reaction, respectively, that (at any dose)-

- Results in death
- Is life-threatening (refers to an event in which the subject was at risk of death at the time of the event; it does not refer to an event which hypothetically might have caused death if it were more severe)
- Requires hospitalisation, or prolongation of existing hospitalisation
- Results in persistent or significant disability or incapacity
- Consists of a congenital anomaly or birth defect

Medical judgement should be exercised in deciding whether an AE/AR is serious in other situations. Important medical events that are not immediately life-threatening or do not result in death or hospitalisation but may jeopardise the patient or may require intervention to prevent one of the other outcomes listed in the definition above, should also be considered serious.

Suspected, Unexpected Serious Adverse Reaction (SUSAR): an adverse reaction that is both unexpected and serious. An adverse reaction is 'unexpected' if its nature or severity is not consistent with the applicable product information (see section 19.2).

Severity (intensity) of Adverse Events and Adverse Reactions

Severity of all AEs and ARs will be graded on a three-point scale of intensity (mild, moderate, severe):

- Mild: Discomfort is noticed, but there is no disruption of normal daily activities.
- Moderate: Discomfort is sufficient to reduce or affect normal daily activities.
- Severe: Discomfort is incapacitating, with inability to work or to perform normal daily activities.

An AE or AR may be severe but not serious

17.2 Expected adverse reactions:

Most adverse events and adverse drug reactions that occur in this trial, whether they are serious or not, will be expected treatment-related toxicities due to the drugs used in this trial. For a full list of expected undesirable effects of CT327, please refer to the Investigator Brochure.

The dose of CT327 to be used in this trial 0.5% as well as a dose of 0.05% have been used previously and were well tolerated by patients. In total 322 patients and 36 healthy volunteers have previously been treated with CT327 ointment and creams. The negative events that have been reported in previous studies include:

Pruritus	6%
Eczema	6%

Headache 5%

Nasopharingitis 3%

Diarrhoea 3%

Application site pruritus 3%

Application site reactions 3%

Rhinitis 2%

Upper respiratory tract infection 2%

Cough 2%

Back Pain 2%

Dermatitis atopic 2%

Dermatitis 1%

Rash 1%

Psoriasis 1%

Fatigue 1%

Vomiting 1%

CT327 has not been detected in the blood of any subjects treated with CT327 cream or ointment.

17.3 Protocol Specifications

For purposes of this protocol:

- All non-serious adverse reactions will be recorded at all trial visits
- Any serious adverse events will be recorded throughout the duration of the trial until the final trial visit.
- Serious adverse events exclude any pre-planned hospitalisations (e.g. elective surgery) not associated with clinical deterioration.
- Serious adverse events exclude routine treatment or monitoring of the studied indication, not associated with any deterioration in condition.
- Serious adverse events exclude elective or scheduled treatment for pre-existing conditions that did not worsen during the trial.
- Serious adverse events exclude increases in tumour size [primary outcome measure, already documented and monitored within trial]

17.4 Recording & Reporting Serious Adverse Events or Reactions:

All adverse events should be reported. Depending on the nature of the event, the reporting procedures below should be followed. Any questions concerning adverse event reporting should be directed to the Chief Investigator, Senior Trial Manager or Trial Manager in the first instance. A flowchart (figure 1) is given below to aid in the reporting procedures.

Adverse Event (including Adverse Reaction): All non-serious adverse events / reactions during drug treatment will be reported on the trial CRF. Severity of AEs will be graded on a three-point scale (mild, moderate, severe). Relation of the AE to the treatment should be assessed by the investigator at site. The Chief Investigator will be responsible for managing all adverse events/reactions.

Serious Adverse Event / Reaction (SAE/SAR, including SUSARs): All SAEs, SARs & SUSARs during drug treatment shall be reported to the Chief Investigator within 24 hours of the site learning of its occurrence. The initial report can be made by completing the serious adverse event CRF and faxing it via the SoHO66 fax to email system which will automatically send email notification to the Chief Investigator and to NCTU. In the case of incomplete information at the time of initial reporting, all appropriate information should be provided as follow-up as soon as this becomes available. Relationship of the SAE to the treatment should be assessed by the investigator at site, as should the expected or unexpected nature of any serious adverse reactions.

The MHRA and main REC will be notified by the Chief Investigator (on behalf of the Sponsor) of all SUSARs occurring during the trial according to the following timelines; fatal and life-threatening within 7 days of notification and non-life threatening within 15 days. SUSARS will be reported using a CIOMS 1 form, specifying the EudraCT number, CTA number, protocol number and trial name, and the data elements listed in Annex 3 of *Detailed guidance on the collection, verification and presentation of adverse reaction reports arising from clinical trials on medicinal products for human use – April 2006*.

All investigators will be informed of all SUSARs occurring throughout the trial on a case-by-case basis.

The Chief Investigator will ensure the Newcastle upon Tyne Hospitals NHS Foundation Trust as Sponsor is notified of any SUSARs in accordance with local trust policy.

Local investigators should report any SUSARs and / or SAEs as required by their local Research & Development Office.

Figure 1

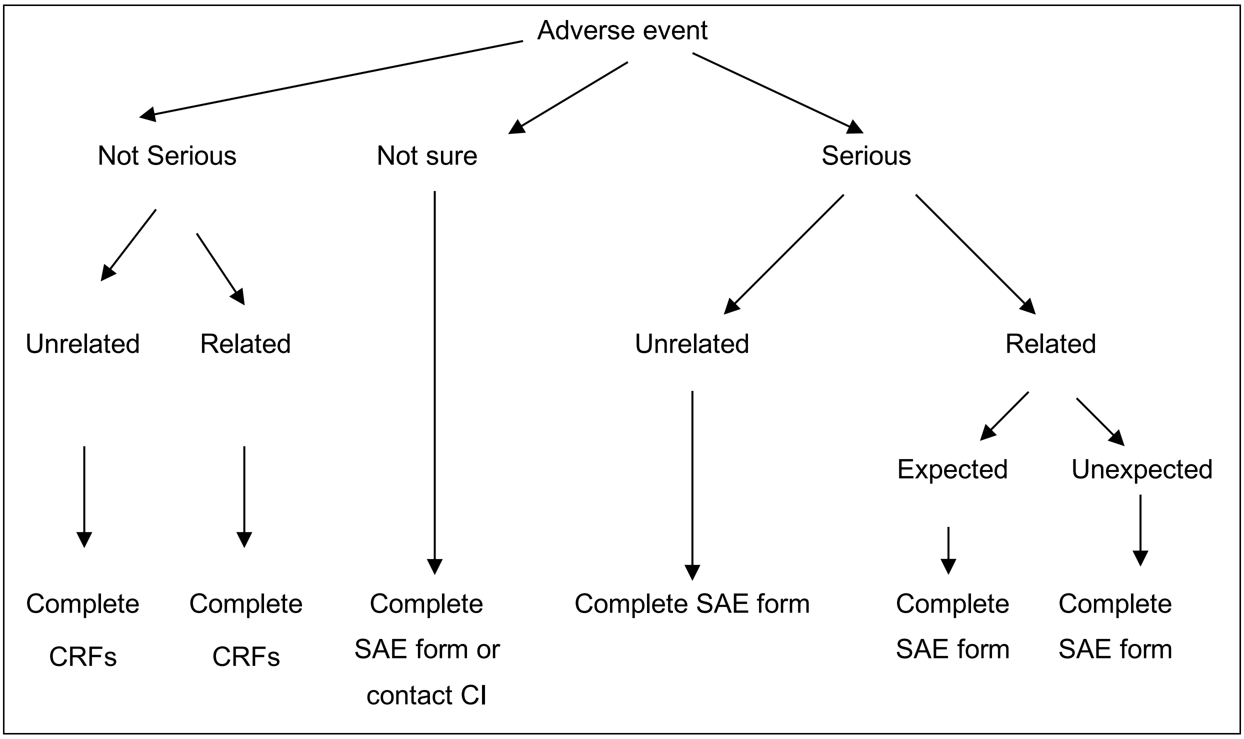

Contact details for reporting SAEs and SUSARs
Please send SAE form(s) via [Fax: **0191 5800 257**]
or
Tel: 0191 208 7623 (Mon to Fri 09.00 – 17.00)

17.5 Pregnancies

If a female participant does become pregnant while participating in the trial, trial drug will be discontinued immediately. Details of the pregnancy should be reported to NCTU within 24 hours of learning of its occurrence. The pregnancy must be followed up to determine outcome. Additional follow-up will no longer be required once the newborn is determined to be healthy.

18. Ethics & Regulatory Issues

The conduct of this trial will be in accordance with the recommendations for physicians involved in research on human subjects adopted by the 18th World Medical Assembly, Helsinki 1964 and later revisions.

Favourable ethical opinion and Clinical Trial Authorisation from relevant Competent Authority (ies) will be sought prior to commencement of the trial. Local approvals will be sought before recruitment may commence at each site. Copies of these approvals will be kept in the Trial Master File at NCTU and in the Investigator Site File at local site.

Information sheets will be provided to all eligible subjects and written informed consent obtained prior to any trial procedures. For subjects who cannot consent for themselves, an appropriate independent witness will provide written consent.

19. Confidentiality

Personal data will be regarded as strictly confidential. To preserve anonymity, any data leaving the site will identify participants by their initials, a unique trial identification code and date of birth. The trial will comply with the Data Protection Act, 1998. All trial records and Investigator Site Files will be kept at site in a locked filing cabinet with restricted access.

All laboratory samples will be labelled with a unique trial identification number and patient date of birth only (linked in anonymised form).

19.1 Safeguarding confidentiality

Data collection and transfer in this trial will comply with NRES and Caldicott guidelines and the Data Protection Act (1998). All patients will be allocated a unique study identifier, which will be used on all eCRFs and paper questionnaires to preserve confidentiality; names or addresses will not appear on completed questionnaires or case report forms. Only a limited number of members of the research team will be able to link this identifier to patient-identifiable details (name & address) which will be held on a separate password protected database in NCTU.

All trial documentation will be held in secure NCTU offices, not open to the public and all members of the research team with access to identifiable or anonymised data will operate to a signed code of confidentiality. Transmission of hard-copy records (e.g. CRFs, questionnaires, SAE reports) between site and the NCTU will be by secure fax, post or hand delivery by the research nurses / trial manager.

Participants will be informed in the patient information sheet about the transfer of information to the NCTU and about levels of access to patient identifiable data, and will be asked to consent to this. Any data used in publications from this trial will be fully anonymised; it will not be possible to identify individual patients from such publications.
Trial data will be entered from source, into a FDA CFR 21part 11 compatible clinical data management software package (MACRO Database system) for processing and management, allowing a full audit trail of any alterations made to the data post entry.
All personal information obtained for the trial will be held securely at the trial site and will be treated as strictly confidential. Site number, screening and randomisation codes will permit the removal of much personal detail (e.g. name and address) prior to transfer of data to the trial office in NCTU.

19.2 Long term data storage

At the end of the trial, original questionnaires, case report forms and consent forms will be securely archived for 15 years following publication of the last paper or report from the trial, in line with Sponsor policy and standard operating procedures. This will also allow any queries or concerns about the data, conduct or conclusions of the trial to be resolved.

20. Insurance and Finance

The Newcastle upon Tyne Hospitals NHS Foundation Trust has liability for clinical negligence that harms individuals toward whom they have a duty of care. NHS Indemnity covers NHS staff and medical academic staff with honorary contracts conducting the trial for potential liability in respect of negligent harm arising from the conduct of the trial. The Newcastle upon Tyne Hospitals NHS Foundation Trust is Sponsor and through the Sponsor, NGS indemnity is provided in respect of potential liability and negligent harm arising from trial management. Indemnity in respect of potential liability arising from negligent harm related to trial design is provided by NHS schemes for those protocol authors who have their substantative contracts of employment with the NHS and by Newcastle University Insurance schemes for those protocol authors who have their substantive contract of employment with the University. This is a non-commercial trial and there are no arrangements for non-negligent compensation.

- Wellcome Trust are funding the trial
- Creabilis are providing CT327 free of charge for the duration of the treatment phase of the trial.

21. Trial Report / Publications

The data will be the property of the Chief Investigator and Co-Investigator(s). Publication will be the responsibility of the Chief Investigator.

It is planned to publish this trial in peer review articles and to present data at national and international meetings. Results of the trial will also be reported to the Sponsor and Funder, and will be available on their web site. All manuscripts, abstracts or other modes of presentation will be led by the TMG and circulated to the Trial Steering Committee and Funder prior to submission. Individuals will not be identified from any trial report.

Participants will be informed about their treatment and their contribution to the trial at the end of the trial, including a lay summary of the results.

22. References

A'Hern, R.P. (2001) 'Sample size tables for exact single-stage phase II designs', *Stat Med.* **20**:859–866.

Bignell, G. R., W. Warren, et al. (2000). 'Identification of the familial cylindromatosis tumour-suppressor gene', *Nat Genet* **25**(2): 160-165.

Draize JH, Woodard G, Calvery Ho. Methods for the study of irritation and toxicity of substances applied topically to the skin and mucous membranes. J Pharmacol Exp Ther Nov 1944 **82**:377-390

Fleming, T.R. (1982) 'One-sample multiple testing procedure for phase II clinical trials', *Biometrics*. **38**:143–151.

Ivanov SV, Panaccione A, Brown B, Guo Y, Moskaluk CA, Wick MJ, *et al*. (2013) 'TrkC signaling is activated in adenoid cystic carcinoma and requires NT-3 to stimulate invasive behavior', *Oncogene*; 8th Aug;**32**(32):3698-710

Jin W, Kim GM, Kim MS, Lim MH, Yun C, Jeong J, *et al*. (2010) 'TrkC plays an essential role in breast tumor growth and metastasis', *Carcinogenesis*. Nov;**31**(11):1939-47.

Rajan, N., R. Elliott, *et al*. (2011) 'Dysregulated TRK signalling is a therapeutic target in CYLD defective tumours', *Oncogene.* 13th Oct;**30**(41):4243-60

Rajan, N., J. A. Langtry, *et al*. (2009) 'Tumor mapping in 2 large multigenerational families with CYLD mutations: implications for disease management and tumor induction', *Arch Dermatol* **145**(11): 1277-1284.

Shabbir, M. and R. Stuart (2010) 'Lestaurtinib, a multitargeted tyrosine kinase inhibitor: from bench to bedside', *Expert Opin Investig Drugs* **19**(3): 427-436.

Skvara, H., F. Kalthoff, *et al*. (2011) 'Topical Treatment of Basal Cell Carcinomas in Nevoid Basal Cell Carcinoma Syndrome with a Smoothened Inhibitor', *The Journal of investigative dermatology.*Aug;**131**(8):1735-44.

Tang JY, Mackay-Wiggan JM, Aszterbaum M, Yauch RL, Lindgren J, Chang K, *et al*. (2012) 'Inhibiting the hedgehog pathway in patients with the basal-cell nevus syndrome', *N Engl J Med*. 7th Jun ;**366**(23):2180–8.

Thiele CJ, Li Z, McKee AE. (2009)'On Trk--The TrkB Signal Transduction Pathway Is an Increasingly Important Target in Cancer Biology', *Clinical Cancer Research.* 30th Sep;**15**(19):5962–7.

23. Appendices

23.1 Appendix A

- **Modified Draize score test**

Score	Grade	Definition
0	Clear	Normal skin surface
1	Almost Clear	Just perceptible erythema and just perceptible papulation/infiltration
2	Mild inflammation	Mild erythema and mild papulation/infiltration
3	Moderate inflammation	Moderate erythema and moderate papulation/infiltration
4	Severe inflammation	Severe erythema and severe papulation/infiltration
5	Very Severe inflammation	Severe erythema and severe papulation/infiltration with oozing/crusting or ulceration

23.2 Appendix B

Patient treatment questionnaire

(Please tick one box for each question)

1. How did you find application of the ointment

- ☐ Easy
- ☐ Neither easy or difficult
- ☐ Difficult

2. How long did it take to apply study treatment?

- ☐ Less than 1 minute
- ☐ More than 1 minute but less than 5 minutes
- ☐ More than 5 minutes but less than 10 minutes
- ☐ More than 10 minutes

3. Overall, how satisfied were you with study medication?

Very satisfied	Satisfied	Neither satisfied or dissatisfied	Dissatisfied	Very dissatisfied
☐	☐	☐	☐	☐

4. If this ointment was available as a treatment would you use it?

- ☐ Yes
- ☐ No

5. <u>Cohort 2 participants only:</u> Which side did you think was the active treatment side? (Please check with the research team if you are uncertain if this question applies to you.)

- ☐ My right
- ☐ My left
- ☐ I don't know

23.3 Appendix C

PATIENT DIARY: Cohort 1

Study Number __ __ __
Patient Initials __ __ __

Day	Ointment Applied? (please circle one)	Any issues or comments?
1	Yes No	
2	Yes No	
3	Yes No	
4	Yes No	
5	Yes No	
6	Yes No	
7	Yes No	
8	Yes No	
9	Yes No	
10	Yes No	
11	Yes No	
12	Yes No	
13	Yes No	
14	Yes No	
15	Yes No	
16	Yes No	
17	Yes No	
18	Yes No	
19	Yes No	
20	Yes No	
21	Yes No	
22	Yes No	
23	Yes No	
24	Yes No	
25	Yes No	
26	Yes No	
27	Yes No	
28	Yes No	

Study Number __ __ __
Patient Initials __ __ __

PATIENT DIARY: Cohort 2

Month	Side	DAY (From ___ / ___ / ___ - To ___ / ___ / ___)																											
		1	2	3	4	5	6	7	8	9	10	11	12	13	14	15	16	17	18	19	20	21	22	23	24	25	26	27	28
For Example		✓	✓	✓	✓	✓	✓	✓ S1	✓ S1	✓ S1	✓	✓	M	✓	✓	✓	✓	✓	✓	W	✓	✓	✓	✓	✓ S2	✓ S2	✓ S2	✓	✓
1	Left																												
	Right																												
2	Left																												
	Right																												
3	Left																												
	Right																												
4	Left																												
	Right																												

Key for completing diary:
✓= Completed as instructed
M = Missed application
W = Wrong cream applied to wrong lesion
S = Symptoms (specify) S1 - ____________________ / S2 - ____________________ / S3 - ____________________ / S4 - ____________________ / S5 - ___________________
O = Other (specify) O1 - ____________________ / O2 - ____________________ / O3 - ____________________ / O4 - ____________________ / O5 - ___________________

Institute of Health and Society Statistics Team

Statistical Analysis Plan for the TRAC Trial

Topical tropomyosin kinase (TRK) inhibitor as a treatment for inherited CYLD defective skin tumours

SAP Version number: 2.0

ISRCTN Number: 75715723

Trial Statistician: Elaine Stamp

Signature: **Date**:

Reviewed and Approved by:

Dr Deborah Stocken, Statistics Co-applicant

Signature: **Date**:

Dr Neil Rajan, Chief Investigator

Signature: **Date**:

This and preceding versions will be stored in the Statistics section of the Trial Master File.

CONTENTS

1. INTRODUCTION 3
1.1 Trial Summary 3
2. TIMING AND REPORTING FINAL ANALYSES 4
3. RECRUITMENT AND RANDOMISATION 4
3.1 Recruitment 4
3.2 Randomisation 4
3.3 Ineligible Patients 4
4. DATA QUALITY 5
4.1 Forms Returned 5
5. STUDY POPULATION 5
5.1 Defining Populations for Analysis 5
5.2 Baseline Patient Characteristics 5
6. TREATMENT RECEIVED 5
7. SAFETY ANALYSIS 6
8. ANALYSES 7
8.1 Definition and Calculation of Outcome Measures 7
8.2 Analyses 8
8.3 Additional Analyses 10
8.4 Decision Criteria 10
8.5 Statistical Software 11
9. STORAGE AND ARCHIVING 11

1. INTRODUCTION

This statistical analysis plan provides guidelines for the analysis and presentation for the analysis of Phase 1b (referred to as cohort 1 in the protocol) and Phase 2a (referred to as cohort 2 in the protocol) of the TRAC trial as per Protocol V2.1 26th August 2015. Unless otherwise stated, each section applies to both phases of the trial. This plan, along with other documents relating to the analysis of this trial, will be stored in the Statistical section of the Trial Master File.

1.1 Trial Summary

This is a non-commercial trial to determine the safety and preliminary efficacy of CT327 in patients with inherited CYLD defective skin tumours.

The trial is split into two phases namely a phase 1b which aims to determine the safety profile of CT327, and a phase 2a that will investigate if CYLD defective tumours respond to CT327.

Patients with germline mutations in a tumour suppressor gene called CYLD develop multiple, disfiguring hair follicle tumours on the head and neck. The prognosis is poor with up to 1 in 4 mutation carriers requiring complete surgical removal of the scalp. There are no effective medical alternatives to treat this condition.

This trial aims to repurpose a topical treatment with the potential to inhibit and possibly prevent tumour growth. Supported by a group of highly motivated patients, this work is driven by experimental data derived from rare, CYLD defective tumours. Whole genome molecular profiling experiments led to the discovery of an attractive molecular target in these skin tumour cells, named TRK.

Creabilis have recently developed an ointment (CT327) containing a TRK inhibitor (mini-pegylated – K252a). CT327 is an attractive, potentially novel agent for this condition as it is applied topically and already has safety data from human Phase 2b trials for the treatment of psoriasis and atopic dermatitis. This trial is designed to determine if the solution of delivering a small molecule to an inherited skin tumour model may represent a safe and feasible treatment for early tumours in these patients.

In Phase 1b the primary objective is to determine the safety of CT327 application in CYLD mutation carriers. The primary outcome will be the number of patients with severe treated skin site reactions as determined by Modified Draize score.

Secondary outcomes of Patient reported quality of life, acceptability of treatment and adverse events within the 4 week treatment period will also be reported.

Phase 2a is a randomised double blind single site trial where the primary objective is to establish if CYLD defective tumours respond to CT327. The primary outcome will be the proportion of tumours responding to treatment by 12 weeks in both actively treated lesions and placebo treated lesions.

Secondary outcome measures will be change in tumour volume, adverse events, compliance, confirmation of the definition of response, expression of targets of TRK signalling in tumour biopsies, patient reported quality of life according to EQ5D and DLQI, acceptability of treatment and a trial specific pain measure.
To achieve the 150 tumours required 15-20 patients will be recruited in Phase 2a.

More detailed information regarding the trial can be found in Protocol V2.1 26th August 2015.

2. TIMING AND REPORTING FINAL ANALYSES

Analysis of Phase 1b data planned at milestone 2 as documented in the trial master file.

Phase 1b: Data entry will be completed by 24/04/2015.

After completion of database reports and checks the data will be released to the trial statistician no later than 13/05/15.

Dummy tables will be populated by and the report circulated approximately a week in advance of the Data Monitoring Committee (DMC) meeting which will take place on 29/05/15.

Final analysis of Phase 2a data is planned with all recruited patients treated and followed for a minimum of 4 weeks at the end of milestone 3, which is 20 months from trial start date, anticipated November 2016, as documented in the trial master file.

Interim analyses will be presented to the Trial DMC planned at the end of phase 1b recruitment and mid-way through phase 2a recruitment, planned at milestone 2, 13 months from start date, anticipated January 2016. Snapshots for data cleaning purposes, without identification of treatment group, may be taken prior to snapshots of data used for reporting. Unless the DMC request increased frequency due to safety concerns. There are no statistical stopping criteria since the trial is not designed around hypothesis testing.

3. RECRUITMENT AND RANDOMISATION

3.1 Recruitment

The following will be reported on:

- Date of snapshot and data-lock (final analysis).
- Report dates when trial opened, last patient entered (interim) and closed to recruitment (final).
- Consort diagram (Appendix 1)
- Plot of recruitment over time.

3.2 Randomisation: Phase 2a only

Randomisation is at the per patient level randomising active and placebo treatments to left or right sided application. There are no stratification factors.
Four or five tumours matched for size will be selected on each side of the patient. Each tumour will be assigned a number to facilitate assessments and then each side of the patient will be allocated randomly to receive either active ointment or placebo ointment.
The number of tumours and unique number of patients recruited will be reported. The number of tumours in each of active and placebo group will be reported.

3.3 Ineligible Patients

Ineligible patients are classed as those patients who are found to subsequently not adhere to the eligibility criteria of the trial. The number of ineligible patients and reasons for ineligibility will be reported. Protocol violators will be reported as part of treatment compliance (section 5.2). Any patients found to be ineligible after entry to the trial will remain in the ITT analysis but may be excluded from the treatment set.

4. DATA QUALITY

4.1 Forms Returned

Data are collected using electronic case report forms (CRF) as detailed in the Data Management Plan (DMP). Completion rates for each CRF will be reported as specified in the DMP. A full listing of CRF's requested are specified in the DMP.

5. STUDY POPULATION

5.1 Defining Populations for Analysis

All patients recruited to Phase 1b will be known as the Phase 1b trial set and to 2a the Phase 2a trial set, as intention to treat sets.

All eligible patients/tumours who start treatment in phase 1b will be known as the Phase 1b treatment set and in Phase 2a the Phase 2a treatment set.

All eligible patients/ tumours who start and complete 12-weeks of treatment in phase 2a will be known as the Phase2a protocol set. If further subsets emerge they will be named appropriately.

5.2 Baseline Patient Characteristics

The analysis sets are the Phase 1b trial set and the Phase 2a trial set.

Baseline patient-level demographic characteristics to be described are gender, ethnicity and age (age to be derived from the date of entry to trial – year of entry to trial- year of birth). Gender and ethnicity will be reported as number and percentage of the trial set and age will be reported as median, Interquartile Range (IQR), Range of the trial set. Please see Appendix 2 for dummy table for baseline characteristics.

Clinical characteristics regarding medical history and concomitant medications will be categorised and tabulated at the patient-level. Tumour location (%) and appearance (hairy or non-hairy) at entry will be reported by randomised group in the Phase 2a trial set at the tumour-level.

6. TREATMENT RECEIVED

Phase 1b will be open label and Phase 2a will be a randomised double blind trial.

In both Phases the dose will be one application in the evening to each tumour as directed at the first visit by the research nurse/doctor. Application will be recorded in a patient diary.

In Phase 1b the participant's will treat their selected tumour with the active treatment for a 4 week period. In Phase 2a all participants will be treating tumours on one half of the body with active CT327 and the other half of the body with placebo according to the randomisation allocation for a 12 week period.

Withdrawals from treatment are cases where the trial drug is discontinued. This may be because the participant decides they no longer wish to continue, cessation of the trial drug is

recommended by the investigator, the participant is pregnant, there is a significant medical condition or an adverse localised or systemic reaction. In both cohorts, participants who withdraw from trial drug prior to the end of treatment visit, will be followed up and included in the analysis, if they are happy to do so.

The analysis sets are the Phase 1b treatment set and the Phase 2a treatment set.

Compliance to the treatment will be reported (Appendix 2) at the patient level as the percentage compliance in terms of days of missed ointment for all tumours. If additionally individual tumours have had missed treatment this will be reported at the tumour level as the number of missed applications (in days) as reported in the diary cards. The total number of applications, at the tumour-level, will be also be reported as median days (IQR, range), over 4 weeks in Phase 1b and over 12 weeks in Phase 2a.

The expected number of applications in a 4-week (28 day) period is 28 and in a 12 week (84 day) period is 84 (+/-3 days dependent on EoT visit date). Compliance to treatment will be summarised as the number of days on treatment (date end-date start) as recorded in the patient diary and reported as median (IQR, Range), as well as the proportion of protocol treatment received, calculated as the number of days of applications as a proportion of the 28/84 day protocol treatment, and reported with 95% Confidence Interval (CI).

The number of patients and reasons for non-compliance will be measured using the patient diaries. The median and IQR of the number of days where ointment was not applied will be reported and any necessary descriptions of the reasons for non-compliance as documented in the comments section of the patient diaries will be reported.

Drop-out of treatment due to pregnancy will be reported.

No significance testing will be carried out.

7. SAFETY ANALYSIS

Most adverse events (AE) and adverse drug reactions that occur in this trial, whether they are serious or not, will be expected treatment-related toxicities due to the drugs used in this trial. For a full list of expected undesirable effects of CT327, please refer to the Investigator Brochure.

AE's are recorded by the clinical staff directly to the database on a three-point scale of severity (mild, moderate or severe) and also by causality (relationship to treatment categorised on a six point scale), as described in the protocol.

The analysis sets are the Phase 1b treatment set and the Phase 2a treatment set.

Any adverse events/reactions from start of treatment and up to the time of tumour excision (Phase 1b) or 4 weeks after end of treatment (Phase 2a), will be categorised according to severity and causality and recorded on the database.

The number of patients (Phase 1b) and tumours (Phase 2a) reporting any AE will be reported clarifying those deemed to be treatment related (Appendix 2).

In Phase 2a, the number of tumours with at least one AE will be reported as a proportion of the total number of tumours. The number of tumours with at least one treatment-related AE, that is those described as definitely, probably or possibly related, will be reported as a proportion of the total number of tumours. The number of tumours with at least one 'moderate' or 'severe' treatment-related AE will be reported as a proportion of the total number of tumours.

The unique number of patients experiencing at least one 'moderate' or 'severe' treatment-related AE will be reported as a proportion of the treatment set.

Proportions will be presented as % (95% CI).

Descriptions of the adverse events may require listing in the text.

No significance testing will be carried out.

8. OUTCOME DATA

8.1 Definition and Calculation of Outcome Measures

8.1.1 Primary Outcome – Phase 1b

The primary outcome measure is the number of patients with severe or very severe treated skin site reactions as determined by the Modified Draize score. The Modified Draize score is defined in the trial Protocol V2.1 26h August 2015.

Modified Draize is collected at the end of treatment. Any score of 4 or 5 categorises the patient as having a severe reaction.

8.1.2 Primary Outcome – Phase 2a

The primary outcome measure is the proportion of tumours responding to treatment by 12 weeks. According to WHO-RECIST criteria, a reduction in 30% volume would be sufficient to classify a "response" to treatment in oncology. In Phase 2a we will use this criteria as a bench mark when determining a response in this patient population at 12 weeks.

Tumour volume will be measured on the predetermined identified tumours in each patient prior to randomisation and measured again and recorded on the same tumours at 4 and 12 weeks. We will have measures of surface area, height and volume taken 3 times at each visit for each tumour. The mean of each set of 3 measurements will be used to determine the percentage change in volume for each tumour to be able to classify tumours as having a response to treatment or not at each time point (Appendix 3).

8.1.2 Secondary Outcomes

Patient Reported Measures

Phase 1b patients will fill in questions 1-4 of the patient treatment questionnaire only, Phase 2a patients will also complete question 5. This is to determine the acceptability of the treatment. This is completed at the final visit, at the end of treatment.

EQ-5D-3L is collected at the baseline visit for both Phase 1b and Phase 2a. The EQ-5D-3L descriptive system comprises the following 5 dimensions: mobility, self-care, usual activities, pain/discomfort and anxiety/depression. Each dimension has 3 levels: no Descriptive problems, some problems, extreme problems. A variable will be derived to describe the unique health of each participant this state is defined by combining 1 level from each of the 5 dimensions. Detailed descriptions regarding calculation of scores can be found in the user guide found at http://www.euroqol.org

The Dermatology Life Quality Index (DLQI) questionnaire is designed for use in adults, i.e. patients over the age of 16 and is a patient reported questionnaire. It is collected at the baseline visit. The DLQI is calculated by summing the score of each question resulting in a maximum of 30 and a minimum of 0. The higher the score, the more quality of life is impaired. The DLQI can also be expressed as a percentage of the maximum possible score of 30. Detailed description of the calculation of the DLQI can be found in the instructions for use on the Cardiff University website. http://www.cardiff.ac.uk/dermatology/quality-of-life

For phase 2a only there are further secondary outcomes of pain assessment. A substantial amendment (Ref: 151395/806479/32/140/42817) was submitted to the MHRA and REC on the 25th June 2015 for the inclusion of the pain assessment forms to phase 2a. Patients were asked at baseline about any pain in existing tumours. At subsequent visits they were asked if the pain had changed and if there were any new painful tumours.

8.2 Analyses of Outcome Data

8.2.1 Analysis of the primary outcome measure: Phase 1b

The number of unique patients with severe reactions will be reported. The primary outcome will be reported as the number of patients with severe reaction in the Phase 1b trial set (and % of patients with associated 95% confidence interval) with a Modified Draize score of 4 or 5.

In addition, median (IQR) Modified Draize scores will be reported.

8.2.2 Analysis of the primary outcome measure: Phase 2a

The analysis set is the Phase 2a trial set.

8.2.2.1. Descriptive analysis

Tumour volumes (mean of the three readings) will be reported descriptively as mean (standard deviation) at baseline by treatment group and overall. Tumour-level change in volume at 4 and 12 weeks will be reported as mean percentage change (with 95% Confidence interval) by randomised treatment group. Graphical representation of percentage change in tumour volume from baseline to 12 weeks will be in the form of a waterfall plot (Appendix 3).

In order to assess the possibility of differing percentage changes being influenced by initial tumour size, the mean percentage change from baseline to 4 weeks and baseline to 12 weeks will be reported by baseline tumour diameter categories. Categories will be determined by quartile measurements of baseline tumour diameter.

Mean volume and mean percentage change will be displayed graphically by treatment group and by baseline tumour diameter category.

8.2.2.2 Primary Analysis of Response

The number of tumours classified as responding to treatment will be reported as a proportion of the trial set with 95%CI at 12 weeks by randomised treatment group. Response to treatment

is classified according to the WHO-RECIST criteria, where a response is either a complete response (disappearance of tumour) or a partial response (decrease in tumour volume of ≥30% at 12 weeks). Non-response is either stable disease (neither complete nor partial criteria met), or progressive disease (increase in tumour volume with no complete response, partial response or stable response reported before increase).

A sensitivity analysis of the Phase 2a primary outcome of response rate will be carried out on the Phase2a 'per protocol' set.

8.2.2.3 Secondary Analysis of Volume measurements

A secondary analysis of the primary outcome measure will be based on a multi-level modelling approach to investigate the relationship between any change in tumour volume and randomised treatment accounting for the three measurements within each tumour as well as multiple tumours within individual patients and multiple measures at baseline and 12 weeks. Random effects multilevel models will account for the nested tumour measurements within both time points and patients, and account for baseline volume measurement and randomised treatment group as covariates. Confidence intervals will be reported.

A repeated measures analysis will look at the mean volume over time at baseline, 4 weeks and 12 weeks accounting for multiple tumours within individual patients including randomised treatment group as a covariate.

8.2.3 Analysis of secondary outcome measures

Secondary outcomes will be analysed using the Phase 1b and phase 2a trial sets separately as Cohort 1 and cohort 2.

8.2.3.1 Other Tumour measures

Tumour measurements of surface area and height will be analysed as described for the primary outcome in 8.2.2 above.

Expression of targets of TRK signalling in tumour biopsies will be analysed and reported graphically.

8.2.3.2 QoL Measures

The Patient Treatment Questionnaire will be analysed descriptively reporting the number of patients answering each response level in each question and reported as a proportion of the treatment set. In Phase 2a the additional question 5 will be reported as the proportion of patients who were able to correctly identify which side had been allocated the treatment ointment. Comments left in the text box will be listed.

Health states determined by the EQ-5D will be reported as numbers (%) of patients in the trial set responding to each dimension. The median (IQR) score for each of the 5 dimensions reported for the Phase 1b trial set and the Phase 2a trial set. The EQ-5D VAS score will be reported as mean and standard deviation (SD).

The mean and SD of the DLQI score will be reported and number and percentage of participants in each scoring band as well as median score and IQR. The analysis set is the trial set.

8.2.3.3 Pain measures

Relating to Phase 2a is the Pain Assessment form. The results will be reported descriptively, separately for Phase 2a active and placebo sets. Number of painful tumours per patient at baseline 4 and 12 weeks, the number of tumours becoming painful during treatment, the number of tumours where pain is reduced during treatment and the number of tumours where pain increases during treatment. Nature of pain will be reported as the number of tumours listed under each pain type and the pain scale will be reported as median range and IQR.

There will be no significance testing.

8.3 Additional Analyses

According to the WHO-RECIST criteria, a 30% reduction in volume is sufficient to classify a "response" to treatment in oncology. In this trial we use this criteria as a bench mark when determining a response in this patient population. As such a further outcome is to consider and confirm a definition of "response" to treatment in this group of patients. We will consider how many tumours are responders, stable or progressive disease at different levels of "partial response". We will report the number of tumours who would be classified as responders for lower levels of reduction in tumour volume at both 4 weeks and 12 weeks. Response rates in the trial set will be presented in tabular form for alternative definitions, based on -5% and - 10% changes in the RECIST definition (Appendix 4).

8.4 Decision Criteria

The decision to progress from Phase 1b to Phase 2a will be based on the number of severe skin reactions reported, with guidance and recommendations from the trial DMC, as reported in the statistics section of the trial protocol and trial master file.

Decision criteria at the end of Phase 2a are predominantly based on the response rate and critical number of responses observed in the treatment group, according to the Fleming A'Hern design. Tumours receiving placebo treatment are not expected to respond to treatment. Using Fleming A'Herns early phase methodology (Fleming 1982, Ahern 2001) any response on the experimental treatment <5% would not indicate a treatment worthy of further investigation. The justification to investigate CT327 further is based on observing a minimum number of responses referred to as the critical number as specified in the document entitled "Critical Number" which is located in the Statistical Trial Master File.

The benchmark for the definition of 'response' was set as 30% reduction in tumour volume given the WHO RECIST oncology criteria. As specified in the protocol, consideration will be given to shifts in this criteria, to smaller reductions in volume, to observe the impact on the response rate and Fleming A'Hern critical number in the treatment group, as outlined in 8.3 above. The reasoning for this exploratory analyses in this early phase clinical trial is to allow further investigation of a biologically interesting treatment which may have not hit a statistical boundary. The conclusions from the trial will be discussed with the DMC and reported with their independent guidance and recommendations.

8.5 Statistical Software

Trial data are input by individual site staff into a MACRO database held and maintained by the Newcastle Clinical Trials Unit.

Data will be extracted from MACRO into statistical software package Stata format. Statistical analyses will be carried out by the Trial Statisticians after data collection for Phase 1b is complete, and after data collection for Phase 2a is complete.

9. STORAGE AND ARCHIVING

The trial database (MACRO) is stored on a hosted server run by Infermed, and backed up on hardware at their hosting partner Rackspace, who are based at a secure location in London. Each Data entry clerk/Trial Manager/Trial co-ordinator is assigned a unique password and certain users have access to their sites data only. Only authorised staff (on the Database Management Team) can grant and have control of access as outlined in SOP DM-001. Any snapshots of the database taken will be kept on the NCTU server which is backed up daily in accordance with SOP DM-006. Once all trial related analysis and activities are completed, the Database will be taken off line, the data will be ‘locked’ and the database will remain password protected. Infermed provide an Archiving module to store the study data and database. Data extracted for sites at the end of the study will be stored on a password protected disc.

Appendix 1

TRAC Phase 2a Consort Diagram – Patient level

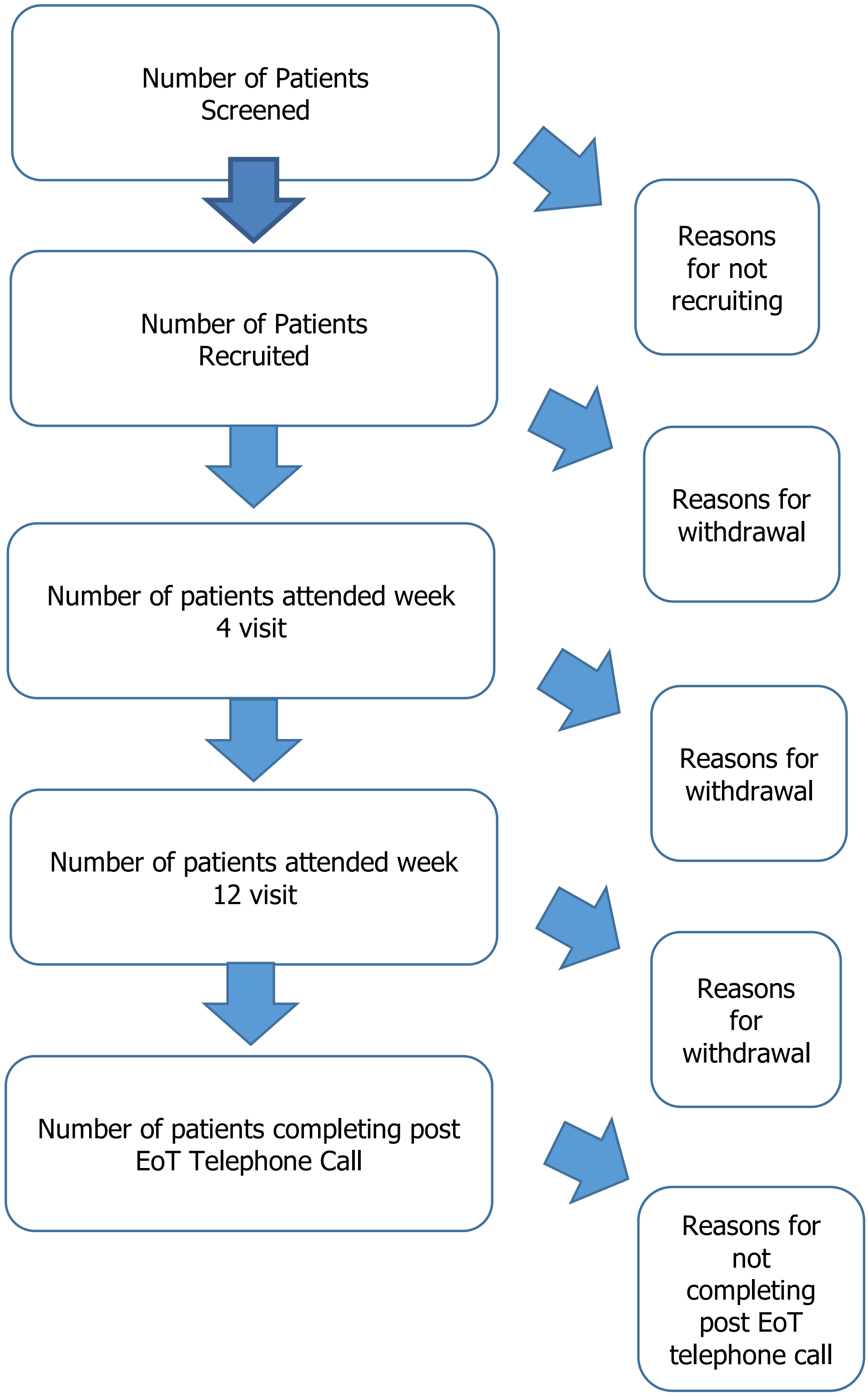

TRAC Phase 2a Consort Diagram – Tumour level

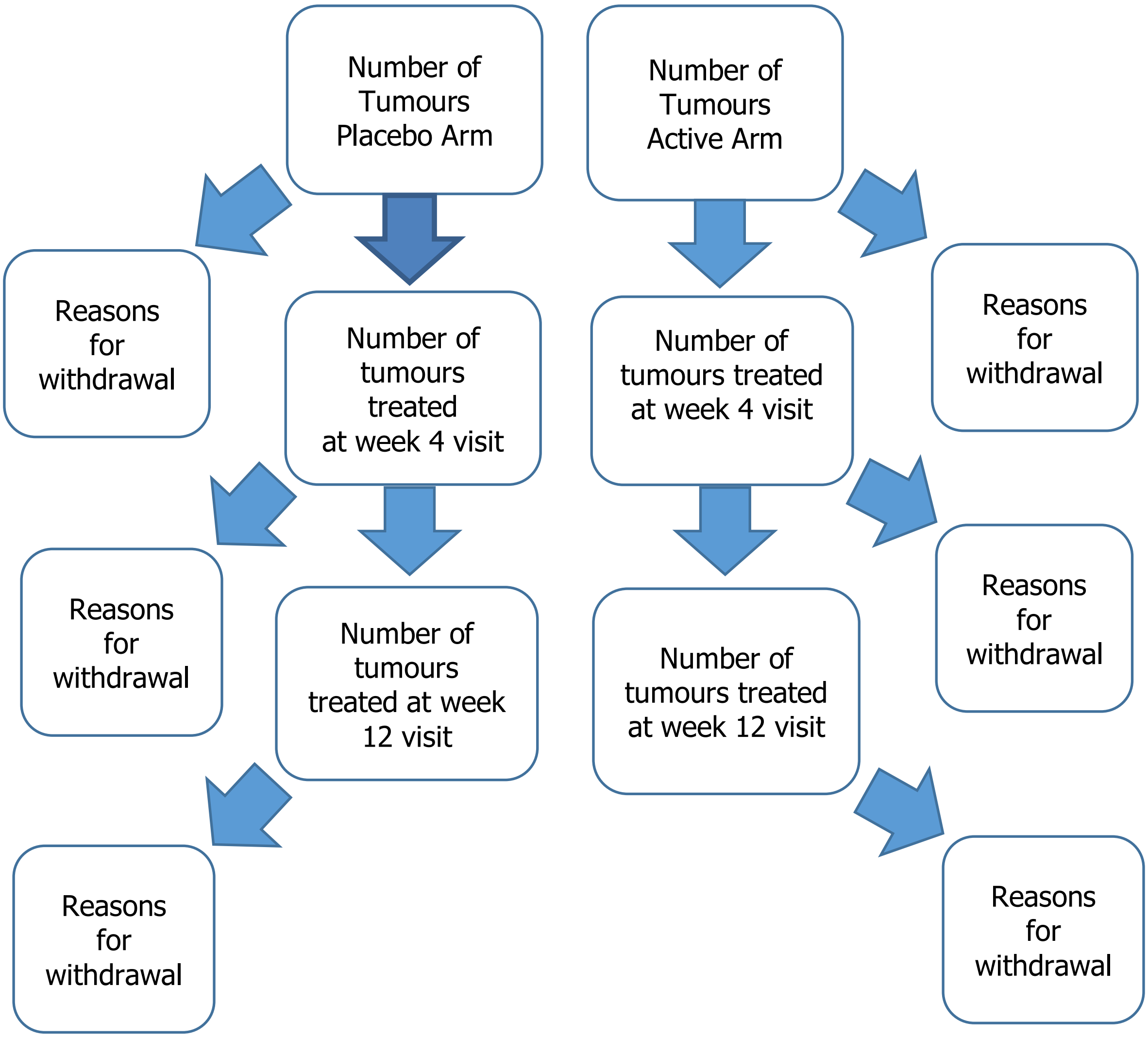

Appendix 2

Baseline Characteristics

Patient Characteristics	n	%
**Gender**<br>Male<br>female		
**Ethnicity**<br>White<br>other		
**Medical Conditions**<br>E.g. Diabetes, cardiac etc.		
**Concomitant Medication**<br>By type, e.g. antihypertensive, analgesics etc.		
**Age**<br>Median<br>IQR<br>Range		

Tumour Characteristics	Active	Placebo	Total
**Tumour size***			
**N**			
**Median**			
**IQR, Range**			
**Location**			
Trunk	N (%)	N (%)	N (%)
Face and Neck			
Scalp			
Limbs			
Other			
**Appearance**			
Hairy			
Non-hairy			
n/a			

*Tumour diameter at baseline, as measured by ruler

TREATMENT RECEIVED/COMPLIANCE

Compliance

**Patients**	**N=**
**Days on treatment**<br>**Median**<br>**IQR, Range**	
**Days missed treatment**<br>**Median**<br>**IQR, Range**	
**% protocol treatment received**<br>**Median**<br>**IQR, Range**	

Tumour Number	Percent missed applications	Active/Placebo

SAFETY ANALYSIS

Adverse Events

Tumours	Active<br>N=	Placebo<br>N=	Total
Any AE<br>Yes*<br>No			
* Severity<br>Mild<br>Moderate<br>Severe			
* Treatment related<br>Yes<br>Definitely<br>Probably<br>Possibly<br>No			

Appendix 3

OUTCOME DATA

**Tumour measures**<br>**N**<br>**Mean (sd)**<br>**Range**	Baseline	4-week	12-week
ACTIVE			
Volume			
Surface Area			
Height			
PLACEBO			
Volume			
Surface Area			
Height			

**VOLUME (cm^3)**	**Active**			**Placebo**			**Overall**		
	**n**	**mean**	**95% CI**	**n**	**mean**	**95% CI**	**n**	**mean**	**95% CI**
Baseline									
Week 4 % change									
Week 12 % change									

Waterfall plot (example)

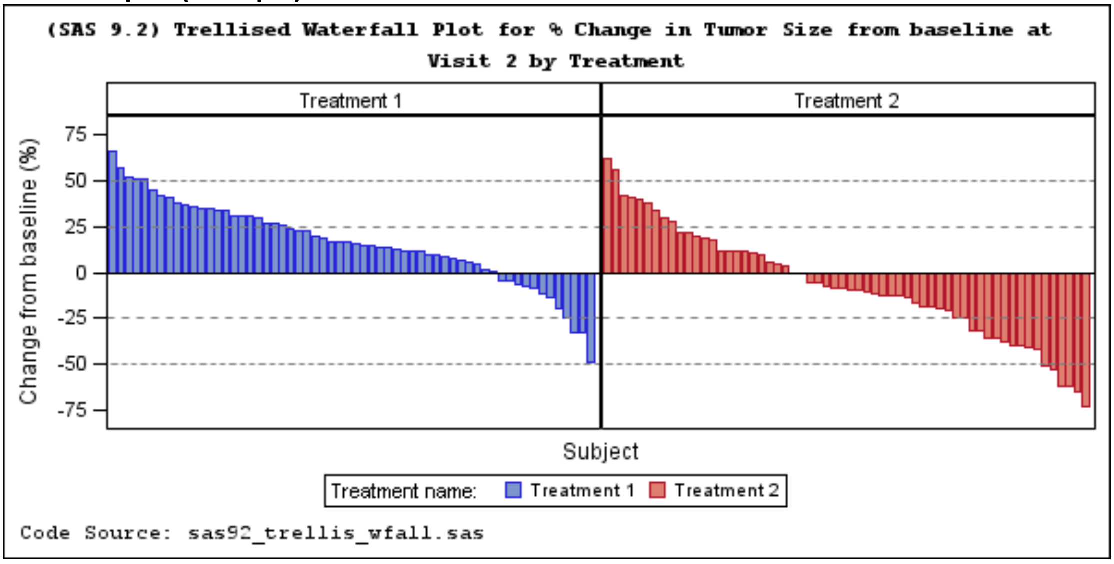

Tumour kinetics

	Active		Placebo	
**Baseline tumour diameter (cm)**	**Mean % change at 4 weeks**	**Mean % change at 12 weeks**	**Mean % change at 4 weeks**	**Mean % change at 12 weeks**
**Quartile 1**				
**Quartile 2**				
**Quartile 3**				
**Quartile 4**				

Analysis of Response

**Tumours**	**Active N=**		**Placebo N=**	
	n (%)	95% CI	n (%)	95% CI
Complete Response				
Partial Response				
**Total Responders**				
Stable Disease				
Progressive Disease				
**Total Non-responders**				

Secondary outcomes

Patient treatment Questionnaire

	ACTIVE Obs	Percent	PLACEBO Obs	Percent
**How did you find the application of the ointment?**				
Easy				
Neither easy or difficult				
Difficult				
**How long did it take to apply study treatment?**				
Less than 1 minute				
More than 1 minute but less than 5 minutes				
More than 5 minutes but less than 10 minutes				
More than 10 minutes				
**Overall, how satisfied were you with the study medication?**				
Very satisfied				
Satisfied				
Neither satisfied or dissatisfied				
Dissatisfied				
Very dissatisfied				
**If this ointment was available as a treatment would you use it?**				

Yes<br>No				

**Which side do you think was the active treatment side?**	Active		Placebo	
	n	% correct	n	% correct
My right				
My left				
I don't know				

EQ-5D

**Dimension**	n	%
**Mobility**<br>I have no problems in walking about<br>I have some problems in walking about<br>I am confined to bed		
**Self-Care**<br>I have no problems with self-care<br>I have some problems washing or dressing myself<br>I am unable to wash or dress myself		
**Usual Activities** (*e.g. work, study, housework, family or leisure activities)*<br>I have no problems with performing my usual activities<br>I have some problems with performing my usual activities<br>I am unable to perform my usual activities		
**Pain/Discomfort**<br>I have no pain or discomfort<br>I have moderate pain or discomfort<br>I have extreme pain or discomfort		
**Anxiety/Depression**<br>I am not anxious or depressed<br>I am moderately anxious or depressed<br>I am extremely anxious or depressed		

**Health States**	**n**	**Percent**
11111		
11121		
12133		
21223		
22231		
Total		

	**Obs**	**Mean**	**Std. Dev.**	**Min**	**Max**
Self-reported health state					

DLQI

Dermatology Life Quality Index	score	n	Percent
Symptoms and feelings	0<br>1<br>2<br>3<br>4<br>5<br>6		
Daily Activities	0<br>1<br>2<br>3<br>4<br>5<br>6		
Leisure	0<br>1<br>2<br>3<br>4<br>5<br>6		
Work and School	0<br>1<br>2<br>3		
Personal Relationships	0<br>1<br>2<br>3<br>4<br>5<br>6		
Treatment	0<br>1<br>2<br>3		

To obtain a total DLQI score the responses are scored as follows with a total score being a maximum of 30:

Very much	scored 3
A lot	scored 2
A little	scored 1
Not at all	scored 0
Not relevant	scored 0

Question unanswered	scored 0
Question 7: “prevented work or studying”	scored 3

The DLQI scores can then be classified as in the table below:

**DLQI (score)**	**Obs**	**Percent**
no effect at all on patient's life (0-1)		
small effect on patient's life (2-5)		
moderate effect on patient's life (6-10)		
very large effect on patient's life (11-20)		
extremely large effect on patient's life (21-30)		
Total		

**DLQI score**	
Median	
IQR	
Range	

Pain Assessment

Numbers of Painful Tumours at baseline

	**Placebo**		**Active**	
	**n**	**%**	**n**	**%**
**Painful tumours**				
**pain length; < 1 month**				
**1-3 months**				
**4-6 months**				
**Over 6 months**				
**No. tumours where pain varied in last week**				
**Pain score median (IQR)**				

Description of Painful Tumours at baseline

	**Placebo**		**Active**	
	**n**	**%**	**n**	**%**
**Throbbing**				
**Hot-burning**				
**Stabbing**				

**Dull**
**Aching**
**Sharp**
**Gnawing**
**Cramping**
**Shooting**
**Tender**
**Heavy**
**Splitting**
**Other**

Numbers of Painful Tumours at week 4

	**Placebo**		**Active**	
	**n**	**%**	**n**	**%**
Painful tumours				
No. tumours where pain increased				
No. tumours where pain decreased				
No. tumours where pain varied during last week				
Pain score median (IQR)				

Description of Painful Tumours at week 4

	**Placebo**		**Active**	
	**n**	**%**	**n**	**%**
**Throbbing**				
**Hot-burning**				
**Stabbing**				
**Dull**				
**Aching**				
**Sharp**				
**Gnawing**				
**Cramping**				
**Shooting**				
**Tender**				
**Heavy**				
**Splitting**				

**Other**

Numbers of Painful Tumours at week 12

	Placebo		**Active**	
	**n**	**%**	**n**	**%**
Painful tumours				
No. tumours where pain increased				
No. tumours where pain decreased				
No. tumours where pain varied during last week				
Pain score median (IQR)				
In the last 3 months;				
No. tumours with more pain				
No. tumours with less pain				
No. tumours about the same				

Description of Painful Tumours at week 12

	**Placebo**		**Active**	
	**n**	**%**	**n**	**%**
**Throbbing**				
**Hot-burning**				
**Stabbing**				
**Dull**				
**Aching**				
**Sharp**				
**Gnawing**				
**Cramping**				
**Shooting**				
**Tender**				
**Heavy**				
**Splitting**				
**Other**				

Appendix 4

Additional Analysis

Reduction in tumour volume	Complete Response	Partial Response	TOTAL RESPONSE	Stable Disease	Disease Progression	Not applicable	Total
≥ 20%	N (%)						
≥ 25%							
≥ 30% (RECIST)							
≥ 35%							
≥ 40%							
