# Supplementary material for: Targeting Tropomyosin Receptor Kinase in Cutaneous CYLD Defective Tumors With Pegcantratinib: The TRAC Randomized Clinical Trial
Source: JAMA Dermatol. 2018 Jun 27;154(8):913–21. doi: 10.1001/jamadermatol.2018.1610 (PMC6128505; doi:10.1001/jamadermatol.2018.1610)
Supplement: Supplement 2. — Materials and Methods eMethods. Supplementary Materials and Methods eTable 1. Medical Conditions and Concomitant Medications in Phase 2a Participants eTable 2. Tumor Characteristics From Participants Recruited to Phase 2a eTable 3. Tumor Measurements of all Tumors at Specified Time Points in Phase 2a Recruited Tumors eTable 4. Impact of Disease on Participants in Phase 1b & 2a as Measured by EQ-5D eTable 5. Impact of Disease on Participants in Phase 1b & 2a as Measured by DLQI eTable 6. Taqman Probes Used for Quantitative PCR eFigure 1. Primary Cultures eFigure 2. Protein Expression [file jamadermatol-154-913-s002.pdf]

Supplementary Online Content

Danilenko M, Stamp E, Stocken DD, et al. Targeting tropomyosin receptor kinase in cutaneous CYLD defective tumors with pegcantratinib: the TRAC randomized clinical trial. *JAMA Dermatol*. Published online June 27, 2018. doi:10.1001/jamadermatol.2018.1610

eMethods. Supplementary Materials and Methods
eTable 1. Medical Conditions and Concomitant Medications in Phase 2a Participants
eTable 2. Tumor Characteristics From Participants Recruited to Phase 2a
eTable 3. Tumor Measurements of all Tumors at Specified Time Points in Phase 2a Recruited Tumors
eTable 4. Impact of Disease on Participants in Phase 1b & 2a as Measured by EQ-5D
eTable 5. Impact of Disease on Participants in Phase 1b & 2a as Measured by DLQI
eTable 6. Taqman Probes Used for Quantitative PCR

eFigure 1. Pegcantratinib targeted 3D cylindroma primary cultures
eFigure 2. pERK and BCL2 protein expression in CYLD defective tumors from TRAC phase 2a.

This supplementary material has been provided by the authors to give readers additional information about their work.

© 2018 Danilenko M et al. *JAMA Dermatology*.

eMethods. Supplementary Materials and Methods

Molecular analyses

Patient genotype Patients in this study carried known pathogenic *CYLD* mutations, and came from well characterised families that have been previously reported.[1-3] Mutations included *CYLD* c.1112C>A (n=2), c.2460delC (n=9) and c.2806C>T (n=2). In two patients, clinical criteria for diagnosis of CCS was confirmed, but no gene mutation was detected, a feature found in 15% or more patients with CCS. [4]

Tissue analysis

Tumor biopsy material was used to determine drug penetration at 3 levels (detailed below) into the punch biopsy. We used a novel method for obtaining material for drug assay from skin tumor cells that were adjacent to sections where we obtained RNA for transcriptomics. We also obtained histology at each of the 3 levels of the tumor to determine how far into the tumor the sample was being obtained from.

Drug penetration assay

Skin punch biopsy samples were serially sectioned such that tissue could be obtained at 3 defined levels within the tumor for drug measurement. The top level contained tissue from approximately 0-300 μm from the surface, the middle level approximately 624-924 μm from the surface and the bottom level approximately 1248-1548 μm from the surface. Samples were analysed using a recently developed and fully validated method for the quantitation of pegcantratinib in human tumor material collected by punch biopsy.[5] The method involves HPLC separation of pegcantratinib followed by in-source fragmentation and detection of a small molecule pegcantratinib-specific

© 2018 Danilenko M et al. JAMA Dermatology.

fragment by LC-MS analysis. The assay was linear over a concentration range of 1-500 ng/mL, with a lower limit of quantification (LLOQ) of 30ng/mL exhibited.

Cell viability assay

Primary cells from patient tumors were cultured on three-dimensional (3D) tissue cullture scaffolds for 28 days and then treated with a range of concentrations of pegcantratinib for 14 days, as previously described.[6] Matched cells were grown on standard tissue culture plastic and treated with pegcantratinib for 3-14 days. Cell viability was assessed using an ATP dependent luminescent assay (CellTitre Glo - Promega UK).

Transcriptomic analyses

RNA was extracted from trial samples and control samples and stranded preparation was performed using the Illumina stranded mRNA kit. Libraries were prepared and sequenced using an Illumina Hiseq 2500, giving 45 million paired end reads per sample which were 100 bp in length. FASTQ files were aligned using the splice aware aligner program STAR to generate alignment files.[7] The read counts for each sample file were counted using the R package Subread.[8] Differential gene expression analysis was carried out using the package DeSeq2.[9] Hierarchical clustering analysis was done employing an Euclidian distance method and a "complete" agglomerative method, using the R package hclust. Assessment of the robustness of clustering was carried out using the R package pvclust, which calculates p-values for hierarchical clustering via multiscale bootstrap resampling.[10] Variant analysis was carried out on the RNAseq data using The Genome Analysis Toolkit (GATK) [11] following GATK best practices guidelines for variant calling on RNAseq. Raw RNAseq reads were mapped against the human reference genome version hg19 using the STAR aligner.[7] The

© 2018 Danilenko M et al. JAMA Dermatology.

GATK tool SplitNCigarReads (specially used for RNAseq) was used to split reads into exon segments and hard-clip any sequences overhanging into the intronic regions. Indel realignment and base recalibration was carried out before variant calling. Variants were screened against the 100K Human Genome Project database. After variant calling, variant filtering was then applied using hard filters.

Quantitative PCR

Quantitative PCR was carried out to validate the expression of *NTRK1, NTRK2 and NTRK3* using established methods. cDNA was generated from an input of 200ng of RNA from snap frozen samples, using the High capacity cDNA Reverse Transcription kit (Applied Biosystems) according to the manufacturer's instructions. PCR reactions were set-up in 25µl reactions using 1µl of input cDNA, Taqman probes (Supplementary Table 5) and Taqman Gene Expression Master Mix (both Applied Biosystems) with qPCR carried out on a 7500 FAST thermocycler (Applied Biosystems) according to the manufacturer's protocol. All samples were run in triplicate and results were analysed using the $2^{-\Delta\Delta Ct}$ method. Water was used as a no template control for each Taqman probe.

Protein expression analyses

Immunohistochemistry (IHC) staining for BCL2 and neurofilament expression and immunoblotting was carried out as described previously.[6] Tissue sections from snap frozen skin tumor biopsies were fixed, blocked and then probed overnight at 4°C with primary antibody against BCL2. Secondary HRP antibodies were applied the following day and visualised with DAB as per manufacturer's instructions (Dako-K4009 Envision). Antibodies against pERK, ERK and BCL2 were obtained from Cell Signalling, USA. Slides were scanned on a Leica slide scanner, and images captured

© 2018 Danilenko M et al. JAMA Dermatology.

from representative areas of each tumor. Image intensity was assessed using Image J calibrated in conjunction with a consultant dermatopathologist.

Statistical Considerations

Phase 1b. Given previous published work, [12,13] it was anticipated that there would be low numbers of treatment related adverse events in phase 1b. It was therefore felt reasonable to recruit only eight patients. Lack of adverse skin reactions in at least five of eight treated patients allowed the trial to progress to phase 2a, following recommendation from an independent Data Monitoring Committee. Escalation of dose was not pursued due to lack of safety data and manufacturing limitations in humans in this exploratory study in CCS.

Phase 2a. It was anticipated that 75 tumors could be measured in each treatment arm (150 tumors in total) in a single centre. As the first exploratory trial, the design parameters are provided as an exemplar of the size of errors that may be anticipated with these patient numbers. Tumors receiving placebo treatment were not expected to respond to treatment, set to be very small at 5% (p0). Using Fleming A'Herns early phase methodology [14] any response on the experimental treatment <5% (p0) would not indicate a treatment worthy of further investigation. A level of efficacy of >15% (p1) would indicate a treatment that warranted further investigation. This level of efficacy was clinically plausible and relevant given that there is no current medical treatment or existing data for this intervention for these patients. 75 tumors recruited in the experimental arm would provide associated error levels of 3.4% type I error (alpha) and 10.8% type II error (beta), deemed acceptable in the early phase setting. The justification to investigate pegcantratinib further is based on observing a minimum number of responses. The trial recruited an equal number of placebo-treated tumors

© 2018 Danilenko M et al. JAMA Dermatology.

to provide an unbiased benchmark. As an early phase trial, statistical analyses were exploratory (not based on hypothesis testing), based on descriptive data presented by treatment group. Small sample trials based on early phase methodologies usually do not have sufficient statistical power for hypothesis driven statistical comparisons and as such are discouraged in favour of point and confidence interval estimation,[15] the strategy followed in this trial.

Supplementary References

1. Nagy N, Rajan N, Farkas K, Kinyó A, Kemény L, Széll M. A Mutational Hotspot in CYLD Causing Cylindromas: A Comparison of Phenotypes Arising in Different Genetic Backgrounds. *Acta dermato-venereologica.* 2013.
2. Rajan N, Langtry JAA, Ashworth A, et al. Tumor mapping in 2 large multigenerational families with CYLD mutations: implications for disease management and tumor induction. *Archives of dermatology.* 2009;145(11):1277-1284.
3. Dubois A, Alonso-Sanchez A, Bajaj V, Husain A, Rajan N. Multiple Facial Trichoepitheliomas and Vulval Cysts. *JAMA Dermatology.* 2017;153(8):826-828.
4. Dubois A, Wilson V, Bourn D, Rajan N. <i>CYLD</i> GeneticTesting for Brooke-Spiegler Syndrome, Familial Cylindromatosis and Multiple Familial Trichoepitheliomas. *PLoS currents.* 2015;7.
5. Zangarini M, Rajan N, Danilenko M, Berry P, Traversa S, Veal GJ. Development and validation of LC-MS/MS with in-source collision-induced dissociation for the quantification of pegcantratinib in human skin tumors. *Bioanalysis.* 2017;9(3):279-288.
6. Rajan N, Elliott R, Clewes O, et al. Dysregulated TRK signalling is a therapeutic target in CYLD defective tumors. *Oncogene.* 2011;30(41):4243-4260.
7. Dobin A, Davis CA, Schlesinger F, et al. STAR: ultrafast universal RNA-seq aligner. *Bioinformatics.* 2013;29(1):15-21.
8. Liao Y, Smyth GK, Shi W. The Subread aligner: fast, accurate and scalable read mapping by seed-and-vote. *Nucleic Acids Res.* 2013;41(10):e108.
9. Love MI, Huber W, Anders S. Moderated estimation of fold change and dispersion for RNA-seq data with DESeq2. *Genome Biol.* 2014;15(12):550.
10. Suzuki R, Shimodaira H. Pvclust: an R package for assessing the uncertainty in hierarchical clustering. *Bioinformatics.* 2006;22(12):1540-1542.
11. Van der Auwera GA, Carneiro MO, Hartl C, et al. From FastQ data to high confidence variant calls: the Genome Analysis Toolkit best practices pipeline. *Curr Protoc Bioinformatics.* 2013;43:11 10 11-33.
12. Creabilis. Investigator's Brochure. In:2015.

© 2018 Danilenko M et al. JAMA Dermatology.

13. Roblin D, Yosipovitch G, Boyce B, et al. Topical TrkA Kinase Inhibitor CT327 is an Effective, Novel Therapy for the Treatment of Pruritus due to Psoriasis: Results from Experimental Studies, and Efficacy and Safety of CT327 in a Phase 2b Clinical Trial in Patients with Psoriasis. *Acta Derm Venereol.* 2015;95(5):542-548.
14. A'Hern RP. Sample size tables for exact single-stage phase II designs. *Statistics in medicine.* 2001;20(6):859-866.
15. Lee JJ, Feng L. Randomized Phase II Designs in Cancer Clinical Trials: Current Status and Future Directions. *Journal of Clinical Oncology.* 2005;23(19):4450-4457.

© 2018 Danilenko M et al. JAMA Dermatology.

Supplementary Tables – Online only

eTable 1. Medical conditions and concomitant medications in phase 2a participants.

eTable 2. Tumor characteristics from participants recruited to phase 2a.

eTable 3. Tumor measurements of all tumors at specified time points in phase 2a recruited tumors.

eTable 4. Impact of disease on participants in phase 1b & 2a as measured by EQ-5D.

eTable 5. Impact of disease on participants in phase 1b & 2a as measured by DLQI.

eTable 6. Taqman probes used for quantitative PCR.

© 2018 Danilenko M et al. JAMA Dermatology.

eTable 1. Medical conditions and concomitant medications in phase 2a participants.

**Medical Conditions**	**No. of patients**
Endocrine disease	5
Skin disease	4
Autoimmune disease	3
Hypertension	3
Mood disorder	3
Respiratory disease	3
Vascular disease	2
Other	21

**Concomitant Medication**	**No. of patients**
Analgesic	9
Antihypertensive	8
Endocrine treatment	4
Sedative	4
Anti-depressant	3
Lipid regulating drugs	3
Proton pump inhibitor	3
Antibiotic	2
Contraceptive	2
Laxative	2
Other	17

© 2018 Danilenko M et al. JAMA Dermatology.

eTable 2. Tumour characteristics from participants recruited to phase 2a.

**Tumour Characteristics**	**Active**	**Placebo**	**Total**
**Tumour size (mm)***			
**N**	75	75	150
**Mean**	4.47	4.00	4.23
**St. Dev**	1.61	1.56	1.59
**Median**	4	4	4
**IQR, Range**	IQR(3,5) R(2,11)	IQR(3,5) R(2,10)	IQR(3,5) R(2,11)
**Location (N %)**	75	75	150
Trunk	5 (6.7)	5 (6.7)	10 (6.7)
Face and Neck	41 (54.7)	39 (52.0)	80 (53.3)
Scalp	29 (38.7)	31 (41.3)	60 (40.0)
Limbs	0 (0)	0 (0)	0 (0)
Other	0 (0)	0 (0)	0 (0)
**Site (N %)**	75	75	150
Hairy	34 (45.3)	36 (48.0)	70 (46.7)
Non-hairy	40 (53.3)	39 (52.0)	79 (52.7)
Scar	1 (1.3)	0	1 (0.7)

© 2018 Danilenko M et al. JAMA Dermatology.

eTable 3. – Tumour measurements in all tumours at all time points in phase 2a recruited tumours.

		n	mean	95% CI	median	IQR
**OVERALL**	height (mm)	150	0.74	(0.68, 0.81)	0.64	(0.47 , 0.91)
Baseline	surface area(mm^2)	150	28.62	(25.78, 31.47)	25.24	(15.50 , 35.78)
	volume(mm^3)	150	7.96	(6.36, 9.56)	4.66	(2.58 , 8.98)
	height (mm)	140	0.78	(0.71, 0.86)	0.66	(0.49 , 0.99)
Week 4	surface area(mm^2)	140	29.20	(26.11, 32.28)	25.47	(15.15 , 37.82)
	volume(mm^3)	140	8.77	(6.80, 10.73)	5.01	(2.44 , 9.16)
	height (mm)	140	0.78	(0.71, 0.86)	0.66	(0.47 , 1.00)
Week 12	surface area(mm^2)	140	29.18	(26.08, 32.28)	25.79	(15.74 , 38.32)
	volume(mm^3)	140	8.71	(6.75, 10.68)	4.80	(2.33 , 9.25)
**ACTIVE**	height (mm)	75	0.74	(0.65, 0.84)	0.65	(0.46 , 0.93)
Baseline	surface area(mm^2)	75	29.19	(25.28, 33.09)	26.49	(15.42 , 38.26)
	volume(mm^3)	75	8.12	(5.92, 10.33)	4.98	(2.57 , 9.40)
	height (mm)	70	0.79	(0.69, 0.90)	0.65	(0.46 , 1.01)
Week 4	surface area(mm^2)	70	30.04	(25.87, 34.22)	27.71	(14.95 , 39.38)
	volume(mm^3)	70	8.99	(6.33, 11.65)	5.12	(2.26 , 11.41)
	height (mm)	70	0.79	(0.68, 0.89)	0.67	(0.46 , 1.02)
Week 12	surface area(mm^2)	70	29.80	(25.63, 33.97)	26.99	(15.57 , 39.06)
	volume(mm^3)	70	8.83	(6.21, 11.47)	4.59	(1.95 , 10.92)
**PLACEBO**	height (mm)	75	0.74	(0.65, 0.83)	0.62	(0.50 , 0.91)
Baseline	surface area(mm^2)	75	28.06	(23.83, 32.29)	24.81	(16.44 , 34.54)
	volume(mm^3)	75	7.79	(5.42, 10.17)	4.38	(2.58 , 8.03)
	height (mm)	70	0.78	(0.67, 0.88)	0.68	(0.50 , 0.96)
Week4	surface area(mm^2)	70	28.35	(23.72, 32.99)	25.24	(15.73 , 34.73)
	volume(mm^3)	70	8.54	(5.59, 11.49)	4.99	(2.48 , 7.90)
	height (mm)	70	0.78	(0.67, 0.89)	0.65	(0.50 , 0.99)
Week 12	surface area(mm^2)	70	28.56	(23.87, 33.25)	25.52	(15.88 , 34.43)
	volume(mm^3)	70	8.59	(5.59, 11.59)	5.01	(2.48 , 8.26)

© 2018 Danilenko M et al. JAMA Dermatology.

eTable 4. Quality of life of participants in phase 1b and 2a as measured by EQ-5D.

Dimension	All Patients (n=16)
**Mobility**	
I have no problems in walking about	14
I have some problems in walking about	2
I am confined to bed	0
**Self-Care**	
I have no problems with self-care	14
I have some problems washing or dressing myself	2
I am unable to wash or dress myself	0
**Usual Activities** (*e.g. work, study, housework, family or leisure activities)*	
I have no problems with performing my usual activities	14
I have some problems with performing my usual activities	2
I am unable to perform my usual activities	0
**Pain/Discomfort**	
I have no pain or discomfort	8
I have moderate pain or discomfort	6
I have extreme pain or discomfort	2
**Anxiety/Depression**	
I am not anxious or depressed	14
I am moderately anxious or depressed	0
I am extremely anxious or depressed	2

© 2018 Danilenko M et al. JAMA Dermatology.

eTable 5. Quality of life of participants in phase 1b and 2a as measured by DLQI.

**Dermatology Life Quality Index**	**Score**	**Patients n=16**
Symptoms and Feelings	0	2
	1	3
	2	2
	3	4
	4	2
	5	3
	6	0
Daily Activities	0	8
	1	6
	2	0
	3	0
	4	1
	5	1
	6	0
Leisure	0	10
	1	3
	2	0
	3	3
	4	0
	5	0
	6	0
Work and School	0	9
	1	4
	2	3
	3	0
Personal Relationships	0	11
	1	4
	2	0
	3	1
	4	0
	5	0
	6	0
Treatment	0	11
	1	5
	2	0
	3	0

**DLQI (score)**	**Obs**	**%**
No effect at all on patient's life (0-1)	3	18.8
Small effect on patient's life (2-5)	6	37.5
Moderate effect on patient's life (6-10)	4	25.0
Very large effect on patient's life (11-20)	3	18.8
Extremely large effect on patient's life (21-30)	0	0.0
**Total**	16	

**DLQI score**	
Median	**4**
IQR	(2, 8)
Range	(0, 15)

© 2018 Danilenko M et al. JAMA Dermatology.

eTable 6. Taqman probe primer details

**Gene**	**Taqman Probe ID**
NTRK1	Hs01021011_m1
NTRK2	Hs00178811_m1
NTRK3	Hs00176797_m1
BCL2	Hs00608023_m1
PUM1	Hs00472881_m1
YWHAZ	Hs01122445_g1

© 2018 Danilenko M et al. JAMA Dermatology.

eFigure Legends

eFigure 1. Pegcantratinib targeted 3D cylindroma primary cultures vs standard cylindroma primary cultures highlights a sensitivity in 3D culture which is associated with upregulation of TRKB and TRKC expression. 3D cylindroma primary cultures were grown for 28 days on tissue culture scaffolds as previously described before being cultured in pegcantratinib for 12 days (4 separate primary tumors, each in triplicate). 2D cylindroma primary cultures were grown for 3 -12 days (4 separate tumors, each in triplicate). 2D cultures tolerated micromolar concentrations of pegcantratinib, whilst 3D cultures had an SF_{50} of 19.2μM. Error bars indicate standard error of the mean.

eFigure 2. pERK and BCL2 protein expression in CYLD defective tumors from TRAC phase 2a. (A) Protein expression of phosphorylated ERK was assessed using SDS-PAGE followed by immunoblotting of lysates generated from tissue sections taken at the top level of biopsied tumors. Quantification of pERK expression was normalised to total ERK expression and the ratio of active:placebo plotted on a bar chart. (B) BCL2 protein expression was determined using sections of the tumor biopsy core taken at the top level. Staining intensity was quantified across 5 regions of each sample, and the mean and standard error of the mean plotted. Seven patients had a statistically significant different level of staining (indicated with an asterisk), between active and placebo sides, although the reduction in BCL2 in active allocated tumors was only seen in 3 patients (1007,1011 and 1013).

© 2018 Danilenko M et al. JAMA Dermatology.

e Figure 1

A

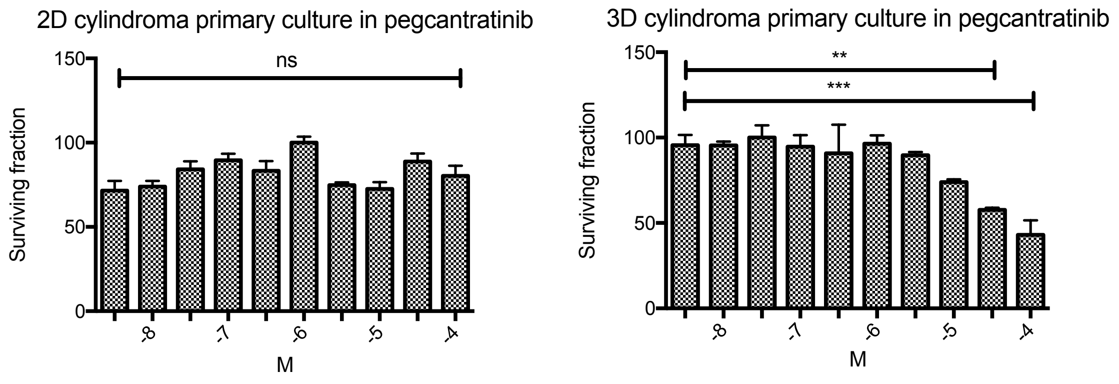

© 2018 Danilenko M et al. JAMA Dermatology.

eFigure 2

A

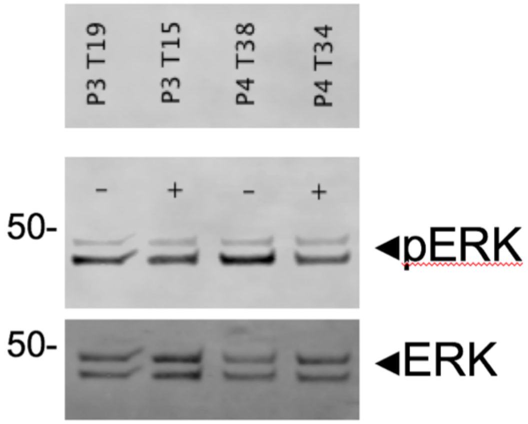

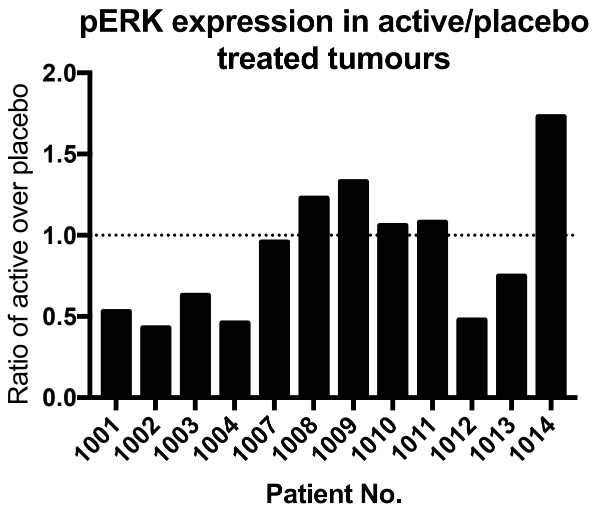

B

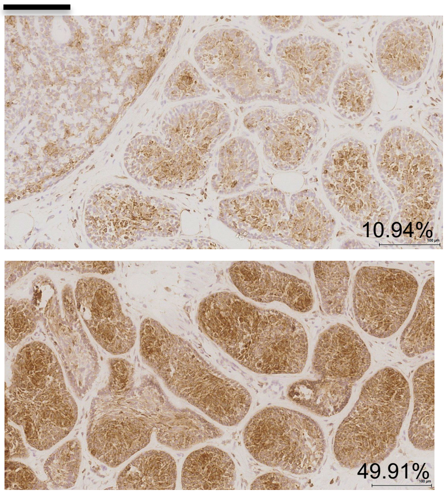

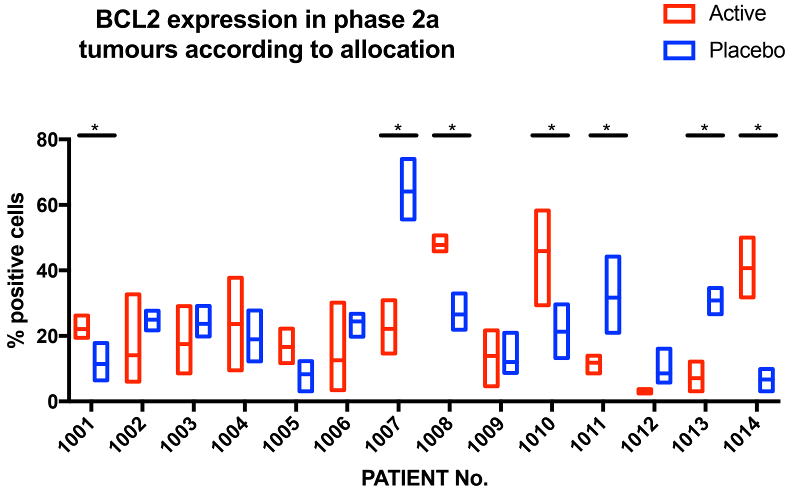

© 2018 Danilenko M et al. JAMA Dermatology.
